# Transfer of 2D Films: From Imperfection to Perfection

**DOI:** 10.1021/acsnano.4c00590

**Published:** 2024-05-29

**Authors:** Phuong V. Pham, The-Hung Mai, Saroj P. Dash, Vasudevanpillai Biju, Yu-Lun Chueh, Deep Jariwala, Vincent Tung

**Affiliations:** † Department of Physics, National Sun Yat-sen University, Kaohsiung 80424, Taiwan; ‡ Department of Microtechnology and Nanoscience, Chalmers University of Technology, Gothenburg 41296, Sweden; § Research Institute for Electronic Science, Hokkaido University, Hokkaido 001-0020, Japan; # Department of Materials Science and Engineering, National Tsing Hua University, Hsinchu 30013, Taiwan; ϕ Department of Electrical and Systems Engineering, University of Pennsylvania, Philadelphia, Pennsylvania 19104, United States; δ Department of Chemical System Engineering, School of Engineering, The University of Tokyo, Tokyo 113-8656, Japan

**Keywords:** 2D films, 2D materials, transfer technology, transfer process, wet transfer, dry transfer, quasi-dry transfer, chemical vapor deposition

## Abstract

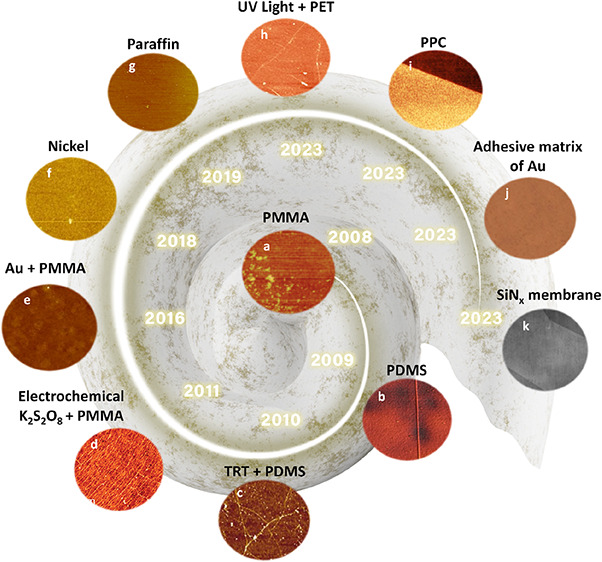

Atomically thin 2D films and their van der Waals heterostructures have demonstrated immense potential for breakthroughs and innovations in science and technology. Integrating 2D films into electronics and optoelectronics devices and their applications in electronics and optoelectronics can lead to improve device efficiencies and tunability. Consequently, there has been steady progress in large-area 2D films for both front- and back-end technologies, with a keen interest in optimizing different growth and synthetic techniques. Parallelly, a significant amount of attention has been directed toward efficient transfer techniques of 2D films on different substrates. Current methods for synthesizing 2D films often involve high-temperature synthesis, precursors, and growth stimulants with highly chemical reactivity. This limitation hinders the widespread applications of 2D films. As a result, reports concerning transfer strategies of 2D films from bare substrates to target substrates have proliferated, showcasing varying degrees of cleanliness, surface damage, and material uniformity. This review aims to evaluate, discuss, and provide an overview of the most advanced transfer methods to date, encompassing wet, dry, and quasi-dry transfer methods. The processes, mechanisms, and pros and cons of each transfer method are critically summarized. Furthermore, we discuss the feasibility of these 2D film transfer methods, concerning their applications in devices and various technology platforms.

## 1 Introduction

Repeatedly, basic discoveries in materials science and surface/interface engineering have ushered in breakthroughs that enhance the quality and performance of semiconductor devices. Most recently this has occurred with two-dimensional (2D) material films that have a van der Waals bonding character out of plane while strong covalent bonding in plane.^1−6^ Ever since A. Geim and K. Novoselov unveiled graphene,^7−13^ a revolution in the search for and fabrication of 2D films at scale has unfolded, sparking strong interest within the fundamental science as well as applied research community.^14−20^ However, the fabrication and integration of 2D films into semiconductor devices at scale still face numerous challenges.^21−28^ One of the primary reasons for this difficulty is the synthesis of high-quality, thin 2D films directly on target substrates. For instance, the growth of most 2D films typically requires high temperatures (>700 °C) using techniques like chemical vapor deposition (CVD) or metal–organic chemical vapor deposition (MOCVD).^29−35^ At such high temperatures, substrates can adversely affect the material growth process. As Khan et al. reported, the growth of graphene on silicon (Si) substrates can lead to unwanted chemical bonding between Si and carbon (C) at 900 °C.^36^ Similarly, Kim et al. demonstrated that boron (B) – oxygen (O) bonds could form during the growth of hexagonal-boron nitrite (h-BN) on silicon dioxide (SiO_2_) substrates.^37^ All these unrelated bonds to 2D film formation can significantly impact the quality and performance of electronic and optoelectronic devices.

As a result, a solution is needed to address the challenge of synthesizing, fabricating, and integrating 2D films onto practical substrates while ensuring good material quality and uniformity. The term “2D film transfer” emerged as a useful solution to this problem.^38,39^ In this approach, after synthesizing 2D films on growth substrates, the transfer method serves as an intermediate solution to detach the 2D films and place them onto target substrates for use in electronic devices. Therefore, research related to transfer methods has gradually gained more attention, especially methods that preserve the quality and minimize surface damage of 2D films.^40−42^ This review aims to provide readers with an overview of widely used transfer methods through recent advances, including wet transfer, dry transfer, and quasi-dry transfer. It will also serve as a valuable reference for scientists to select and evaluate the pros and cons of various 2D film transfer techniques.

**Figure 1:**
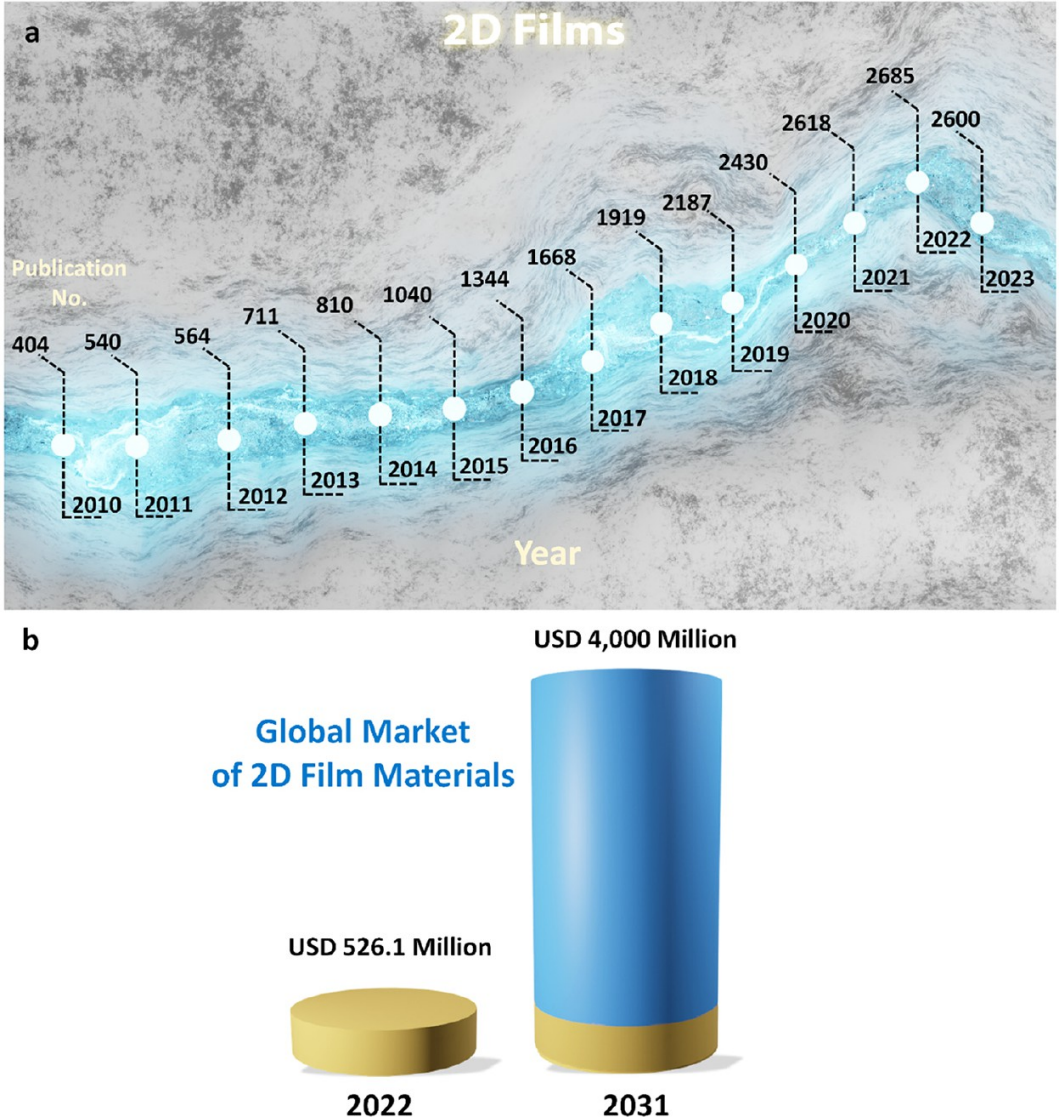
a) The annual publications of “2D Films” (2010–2023) (Source: Web of Science), and b) Global market forecast of 2D film materials via the revenue in 2022 and 2031. (Source: www.researchandmarkets.com) (Access date: 30/10/2023).

**Figure 2:**
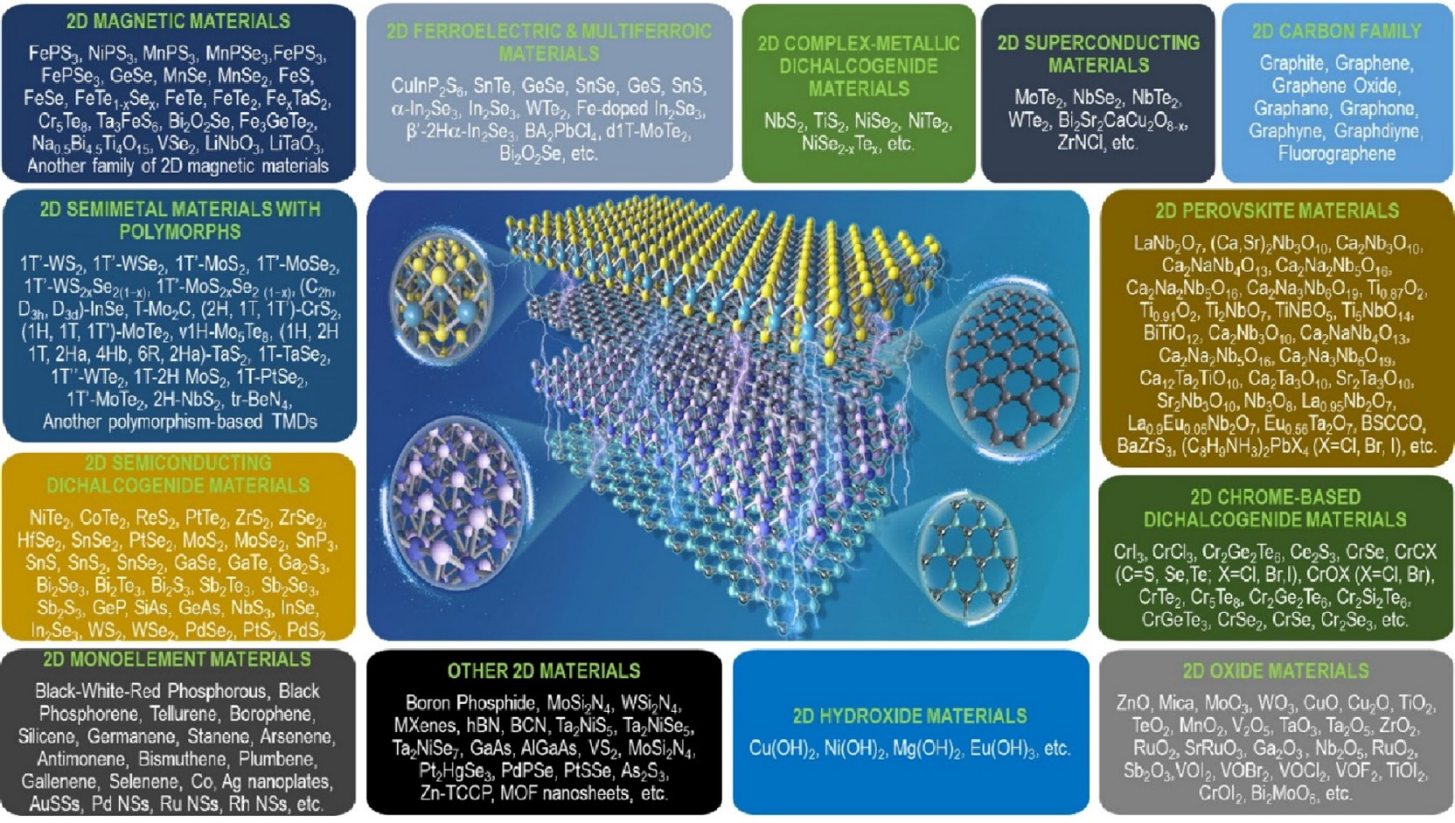
An emerging grand family of 2D films that has been discovered in both experimental synthesis and theoretical computation in the 21st century. Reproduced with permission from ref (3). Copyright 2022 American Chemical Society.

Figure 1a reveals the evolution flow through the annual publications from 2010 to 2023. Drastically, it reached up to 5229% (from 404 in 2010 and 21,126 in 2023). Regarding the forecast from Research and Markets (www.researchandmarkets.com) for the global market of 2D film materials, the rapid growth via the revenue in 2022 and 2031 are 526.1 and 4,000 million US$, respectively (Figure 1b). It potentially depicts the compound annual growth rate (CAGR) of 25.3% during this time. Meanwhile, Figure 2 reveals an emerging grand family of 2D films that has been discovered in both experimental synthesis and theoretical computation recently.^3^ It will certainly be a perfect arrow pointing the way for multidisciplinary scientists to seek and create more breakthroughs in this hot field.

## 2 Transfer Strategies of 2D Films

**Figure 3:**
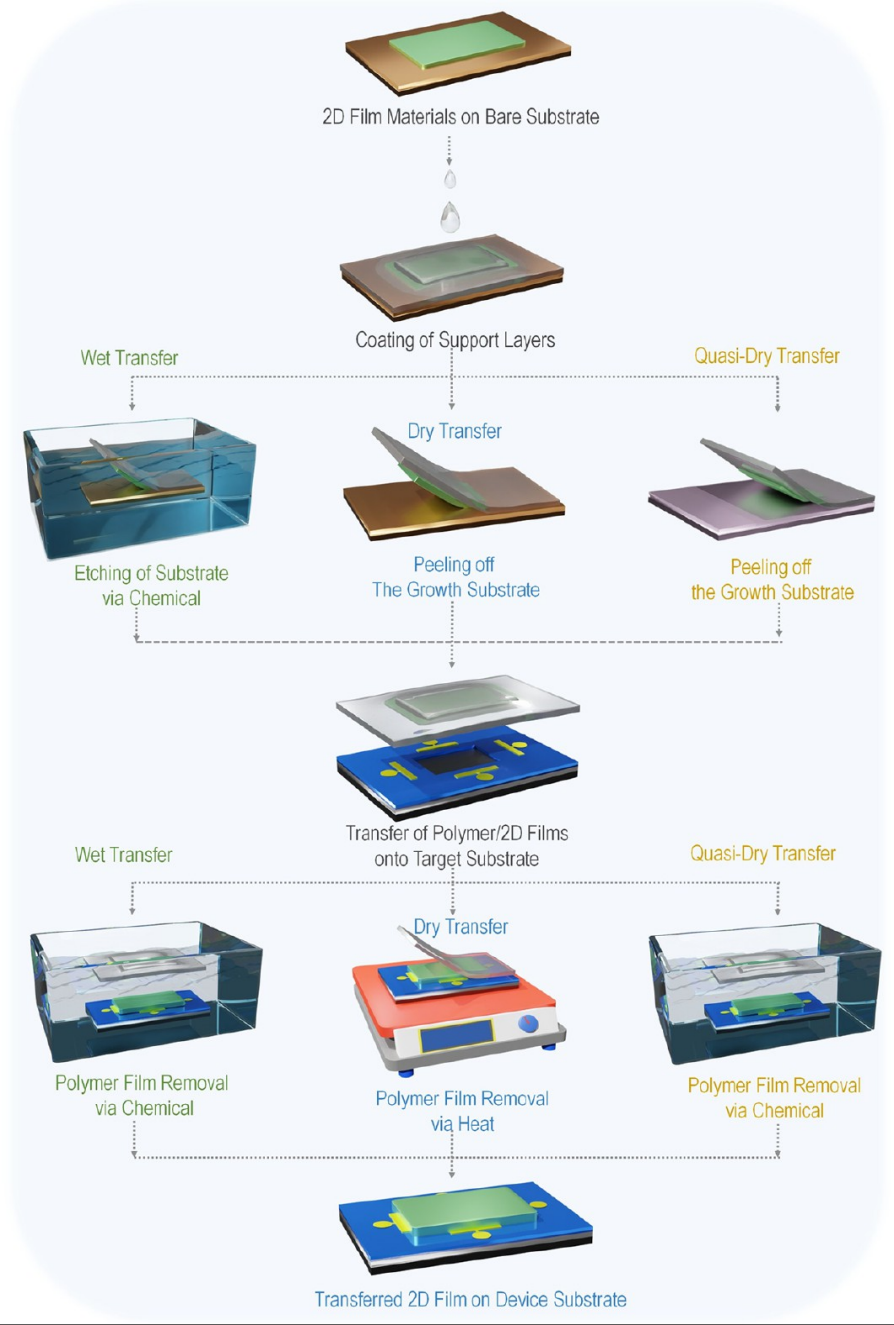
An overview of 2D film transfer technologies on devices, including wet, dry, and quasi-dry transfer.

Transfer methods are pivotal in achieving the extraordinary properties of atomic-sized 2D films when applied to semiconductor devices.^43−45^ Typically, this process involves detaching the 2D films from their growth substrate and transferring them onto a target substrate for further device integration or characterization.^46^ The transfer process often employs a support layer coated onto the 2D films to provide mechanical stability. Subsequently, the original growth substrates can be removed chemically or mechanically through various methods, leaving the 2D films attached to the support layers. Finally, these 2D films are placed onto the desired substrates, and the support layer is removed, completing the transfer process. However, each method has distinct implications for removing the growth substrate, or the support layer, and minimizing contamination and damage to the material’s surface, making it crucial to select the appropriate technique. Figure 3 shows the fundamental step-by-step process of wet, dry, and quasi-dry transfer of 2D films.

### 2.1 Wet Transfer

The wet transfer process is a commonly employed method in transferring 2D films closely linked to the utilization of a liquid or solvent medium, such as water or a chemical solution.^47,48^ This liquid not only aids in separating the 2D films from its source substrate but also offers valuable mechanical support during the transfer process. In this subsection, we present several strategies to promote and improve the removal efficiency of 2D films. Table 1 lists reports on the transfer and application 2D films that used such wet transfer methods.

**Table 1:** Wet-Transfer Strategies on Representative 2D Films and Related Applications^a^

2D Films	Support layer	2D Films Size	Applications	ref
Graphene	Poly(methyl methacrylate) (PMMA)	∼ 1 cm^2^	Field-effect transistors (FETs)	(49)
Graphene	Polyimide (PI)	∼ 0.15 cm^2^	Micro supercapacitors	(50)
Graphene	PMMA	4 cm^2^	–	(51)
Graphene	PMMA	**–**	–	(52)
Graphene	PMMA	a few tens μm	Polymer solar cells	(53)
Graphene	Poly(9,9-di*n*-octylfluorene -*alt*-(1,4phenylene-(4-*sec*butylphenyl) imino)-1,4- phenylene)/PMMA stamp	**–**	Organic Light- Emitting Diodes (OLEDs)	(54)
Graphene	Polydimethyl s-iloxane (PDMS)	4 cm^2^	–	(55)
Graphene	Paraffin	40 × 20 mm^2^	Field-effect transistors (FETs)	(56)
Graphene	PMMA	–	FETs	(57)
Tungsten selenide (WSe_2_)	PMMA	30–50 μm	Nanogenerators	(58)
WSe_2_	PMMA	–	Photodetectors	(59)
WSe_2_	Varnish	15 × 15 mm^2^	Ferroelectric FETs	(60)
Tungsten disulfide (WS_2_)	PMMA	∼1 cm^2^	–	(61)
WS_2_	PMMA	∼1.5 cm^2^	Back-gate FETs	(62)
WS_2_	Polyvinyl alcohol (PVA)	–	Photodetector	(63)
WS_2_	Polystyrene (PS)/PMMA	∼ 50 μm	Photodetector	(64)
WS_2_	PMMA	**–**	Straintronics	(65)
Molybdenum disulfide (MoS_2_)	PMMA	**–**	–	(66)
MoS_2_	PDMS	**–**	Light-Emitting Diode (LED)	(67)
MoS_2_	PMMA	∼5 × 10 cm^2^	Ultrastable Transistors	(68)
MoS_2_	Polypropylen e Carbonate (PPC)	∼30–40 μm	Transistors	(69)
MoS_2_	PMMA	**–**	Photocatalytic	(70)
MoS_2_	PS	**–**	–	(71)
MoS_2_	Epoxy glue	1 cm^2^	Flexible FETs	(72)
MoS_2_	PS	1 cm^2^	Photodetector	(73)
MoS_2_	PMMA	**–**	Organic Solar cells	(74)
h-BN	PMMA	5 × 5 mm^2^	FETs	(75)
h-BN	PS	**–**	LED	(76)
h-BN	Poly[4,5difluoro-2,2bis(trifluoro methyl)–1,3dioxole-*co*- tetrafluoroeth ylene (PTFE)	**–**	FETs	(77)
h-BN	Nafion and Sulfonated poly(ether ether ketone) (SPEEK)	4 × 4 cm^2^	Batteries	(78)
Niobium diselenide (NbSe_2_)	PMMA	**–**	FETs	(79)
Black Phosphorus (BP)	PMMA	**–**	Photodetectors	(80)
BP	PMMA	**–**	Phototransistor	(81)

a Here, “–” means “not applicable”.

#### 2.1.1 Polymer-Assisted Transfer

##### 2.1.1.1 PMMA Transfer

**Figure 4:**
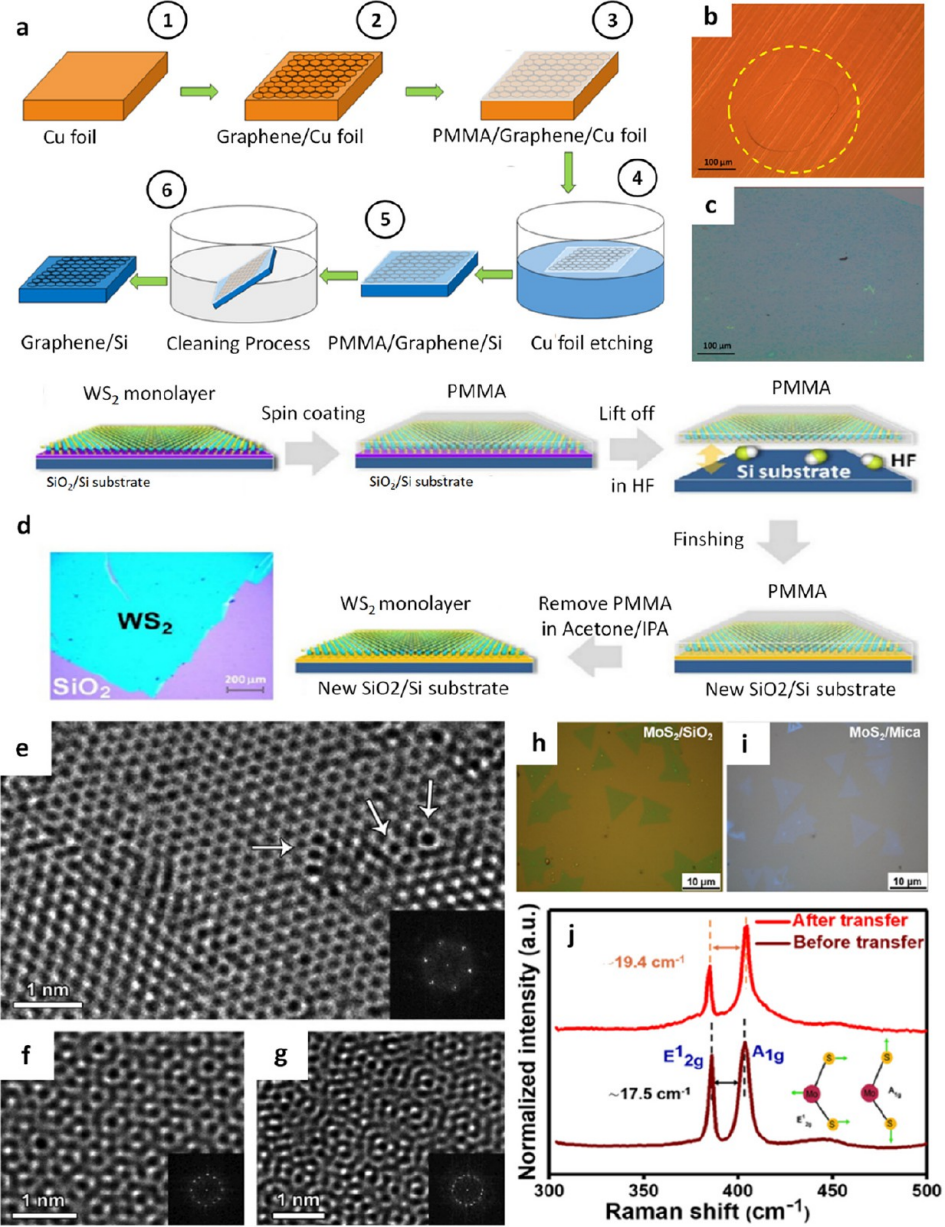
PMMA-assisted transfer of graphene, WS_2_, and MoS_2_. a) Various stages encompassed in the wet chemical transfer process of graphene. b) Cu foil after graphene growth, and c) graphene transferred on Si/SiO_2_ substrate. a-c) Reproduced with permission from ref (52). Copyright 2019 American Chemical Society. d) Schematic representation of the PMMA-assisted transfer method onto different substrates. e-g) High-resolution transmission electron microscopy (HRTEM) images of WS_2_ film. Insets show the fast Fourier transformation (FFT) of the corresponding TEM micrograph. e) A WS_2_ film displaying both crystalline areas and certain imperfections, such as enlarged rings, indicated by arrows. f) and g) bilayer and trilayer WS_2_ exhibiting distinct stacking arrangements, as evidenced by the Moiré pattern formed and verified through FFT analysis. d-g) Reproduced with permission from ref (61). Copyright 2013 American Chemical Society. h) Visual representations of MoS_2_ on SiO_2_/Si substrate before the transfer process and (i) mica substrate after transfer. k) Raman spectra illustrating MoS_2_ characteristics before and after the transfer. h-k) Reproduced with permission from ref (66). Copyright 2020 American Chemical Society.

**Figure 5:**
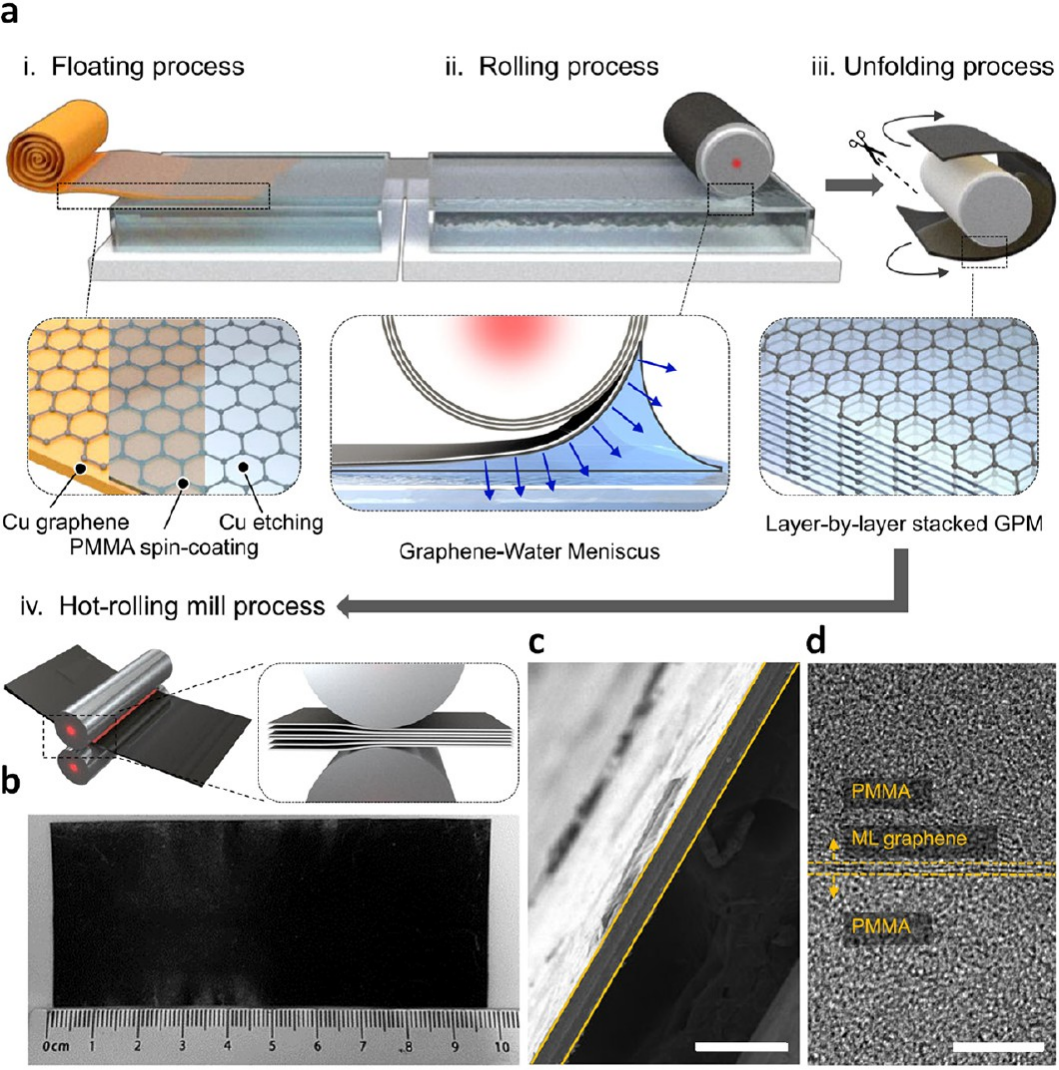
PMMA and Rolling-up-assisted transfer of graphene. a) Illustration of the float-stacking procedure for the graphene-PMMA laminate:(i) Floating the graphene-PMMA membrane on a DI water bath subsequent to wet-etching of the bottom Cu foil, (ii) Layer-by-layer stacking of graphene-PMMA membranes through a rolling process,(iii) Cutting and unfurling of the stacked-graphene-PMMA membrane, and (iv) Hot-rolling mill treatment of the stacked-graphene-PMMA membrane. b) Image capturing the graphene-PMMA laminate postpreparation, consisting of 100 layers of graphene-PMMA membrane. c, d) Cross-sectional SEM and TEM visuals of the graphene-PMMA laminate-100. Monolayer graphene is embedded within the PMMA matrix, devoid of structural flaws. Scale bar: 100 and 5 μm, respectively. Reprinted with permission under a Creative Commons (CC BY 4.0) License from ref (85). Copyright 2024 Nature Publishing Group.

The polymer-assisted transfer technique relies on the adhesion and interaction between the material and polymer. This polymer is fully coated onto the surface of the growth substrate, creating a flat and stable platform that securely holds the delicate 2D films in place. This ensures the material’s integrity and prevents it from folding or wrinkling during the transfer process. In most studies employing this method, PMMA is perhaps the most widely used polymer.^82^ This preference is primarily due to PMMA’s exceptional solubility in various solvents, including acetone. This solubility makes PMMA an excellent choice for creating a supportive polymer layer during wet transfer. Furthermore, the solubility of PMMA allows for the easy removal of the sacrificial PMMA layer once the transfer is completed. In a report, Kashyap et al. have transferred graphene from copper (Cu) foil to Si/SiO_2_ via a protection layer PMMA (Figure 4a).^52^ Here, Cu foil was etched by diluted nitric acid solution, and PMMA was removed by acetone. Graphene can be observed through optical images after it is transferred to Si/SiO_2_ substrate, showing a clean, continuous, and wrinkle-free transfer of graphene on the substrate (Figure 4b, c). In another publication, Elias et al. obtained a few layers and a single layer of WS_2_ and transferred them to the Si/SiO_2_ substrate (Figure 4d).^61^ Although the sample has a fairly high quality, a few defects can be observed (Figure 4e). During the transfer process, WS_2_ films could be folded or wrinkled, due to this fact, it can also find some regions with different WS_2_ thickness and stacking. Furthermore, the authors noted the presence of Moiré patterns and confirmed the varied stacking order in the bilayer and trilayer WS_2_ through respective fast Fourier transforms (Figure 4f, g). In a similar report by Sharma et al., PMMA as a support layer for the transfer process of MoS_2_ onto mica substrate has also been applied.^66^ The positive outcomes were gathered, and it was concluded that there was no any polymer residue, cracks or winkles on surface of MoS_2_ after transfer (Figure 4h, (i). In order to assess and validate the crystalline quality, Raman analyses were conducted (Figure 4j). In the case of as-grown MoS_2_ on SiO_2_/Si, the authors detected Raman peaks at approximately 386.1 cm^–1^ (E^1^_2g_) and 403.66 cm-1 (A_1g_), whereas for transferred MoS_2_, the E^1^_2g_ mode was observed at around 385.0 cm^–1^ and the A_1g_ mode at 404.4 cm^–1^. For the monolayer MoS_2_, there was a slight change in Δω from 17.5 to 19.4 cm^–1^ after transfer. The strain state of MoS_2_ undergoes alteration pre and post-transfer, resulting in a red-shift in the E^1^_2g_ mode and a blue-shift in the A_1g_ mode. This phenomenon can be elucidated by the fact that the E^1^_2g_ mode is relatively insensitive to substrate material, whereas the A_1g_ mode exhibits significant sensitivity to substrates, showcasing considerable stiffening.^83,84^ Additionally, the suggested procedure enables the recycling of the growth substrates, rendering it a cost-effective process. In another approach, Kim et al. undertook the transfer of graphene by combining it with PMMA and leveraging the support of the Roll-to-roll process (Figure 5a-d).^85^ This method facilitated the production of multifunctional composites with precisely controlled layers and spacing of the semi-infinite graphene reinforcement within the polymer matrix. Consequently, the authors observed a significant enhancement in the mechanical and thermal properties of the graphene-PMMA laminate. The floating-stacking strategy holds great promise in realizing functional nanocomposites based on low-dimensional nanomaterials, which serve as ideal semi-infinite reinforcements that are challenging to disperse.

##### 2.1.1.2 PDMS Transfer

**Figure 6:**
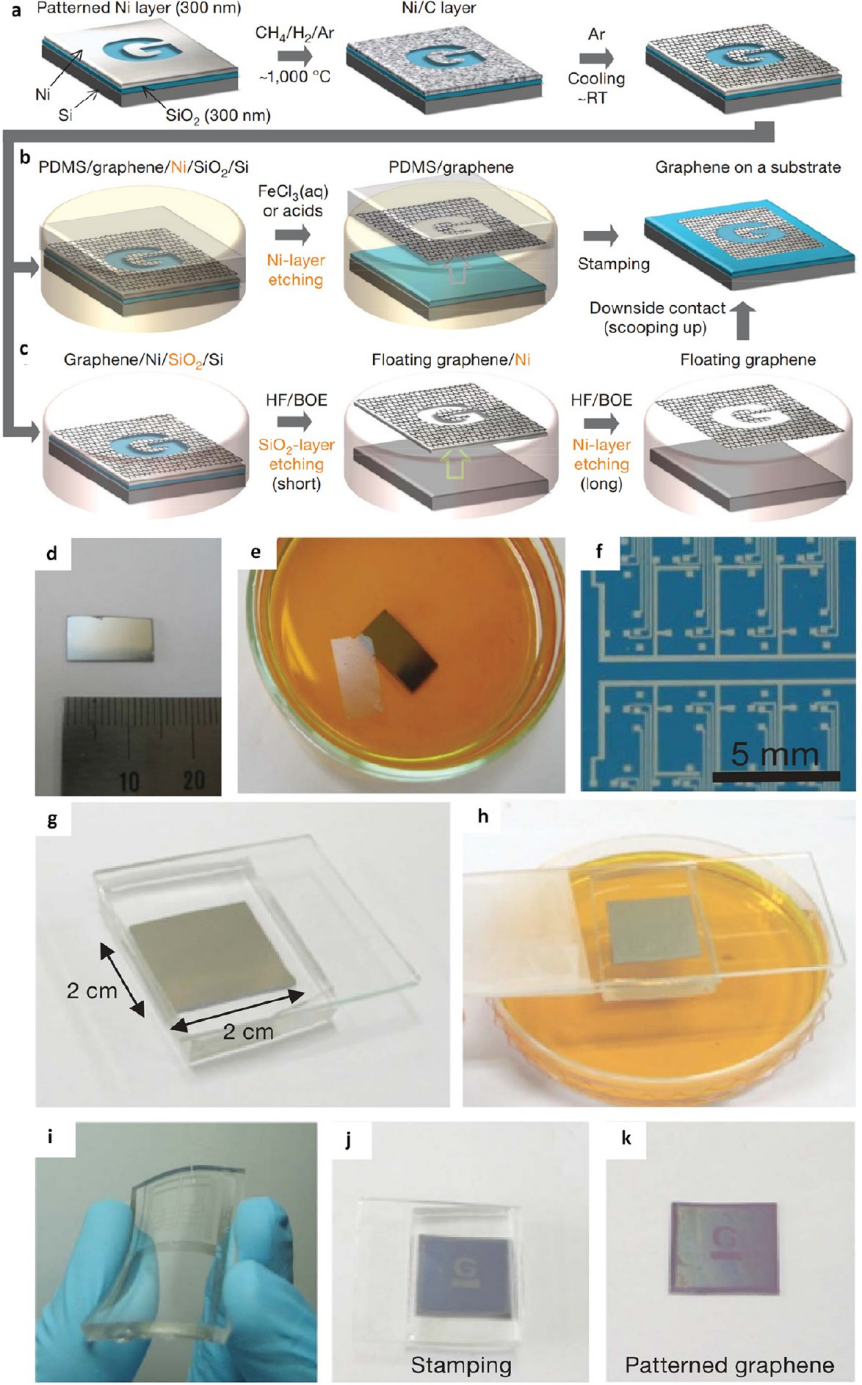
PDMS-assisted transfer of graphene. a) Fabrication of graphene films with defined patterns on thin nickel layers. b) Employing FeCl_3_ for etching and transferring graphene films utilizing a PDMS stamp. c) Utilizing BOE solution for etching and transferring graphene films at room temperature. d) Achieving centimeter-scale graphene film growth on a Ni/SiO_2_/Si substrate. e) Obtaining a floating graphene film post-Ni-layer etching can be directly transferred by contacting substrates. f) Synthesizing graphene films of various shapes on top of structured Ni layer. g) Attaching the PDMS substrate to graphene, and h) removing the underlying Ni layer using FeCl_3_ solution. (i) Demonstrating transparency and flexibility in graphene films on PDMS substrates. j) and k) Ensuring conformal contact between the PDMS stamp and a SiO_2_ substrate. a-k) Reproduced with permission from ref (55). Copyright 2009 Nature Publishing Group.

While PMMA is relatively easy to remove during the transfer process, it can be prone to wrinkles, folds, and cracks in some instances due to its limitations in compatibility and flexibility. These challenges make PMMA less suitable for large-scale materials applications. A study by Kim et al. explored an alternative by replacing PMMA layers with PDMS, known for its low toxicity, softness, nonstick properties, and low surface energy.^55^ The authors achieved large-scale pattern growth of graphene films by transferring them to a target substrate. This was done after removing the initial support layer using iron(III) chloride (FeCl_3_) and buffered oxide etchant (BOE) (Figure 6a-c). The authors meticulously observed each step of the transfer process, as illustrated in Figure 5d-k, and based on the results obtained, the authors believed that the introduced ripples enhanced the stability of graphene films against mechanical stretching, rendering them more expandable. Opting for multilayer graphene samples demonstrated a benefit in terms of mechanical strength, supporting large-area film structures, whereas thinner graphene films exhibited increased optical transparency. This technique showcased a simple method for transferring large-scale, high-quality, stretchable graphene films employing CVD on a nickel (Ni) layer. The patterned films could be effortlessly transferred to a stretchable substrate through a straightforward contact process.

PMMA and PDMS are commonly chosen materials in 2D film transfer studies due to their ease of use. However, controlling potential contamination and the impact of chemical reactions during polymer layer removal remains challenging for both materials.^86,87^ These challenges can lead to quality issues with 2D films and compromise their device performance. Therefore, finding alternative materials for the transfer process with more excellent stability and reliability is a current and significant research focus.

##### 2.1.1.3 Paraffin Transfer

**Figure 7:**
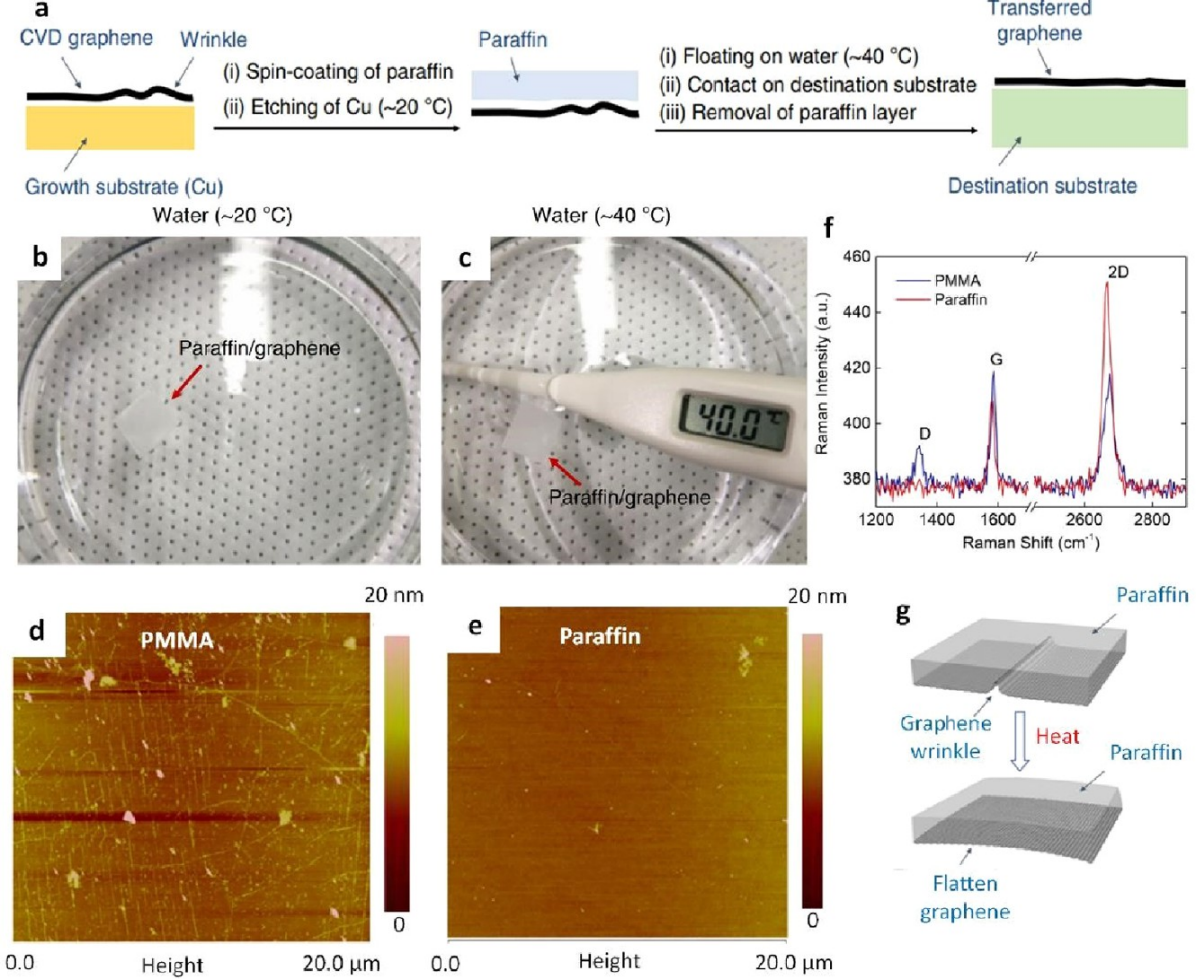
Paraffin-assisted transfer of graphene. a) Diagram illustrating the procedure for transferring graphene with the assistance of paraffin. b) Illustrations portraying the impact of paraffin’s thermal expansion on the wrinkling of graphene. c) Images capturing a representative graphene film supported by paraffin, floating on water at various temperatures, confirming that the paraffin layer remains in a solid state at approximately 40 °C. d) atomic force microscope (AFM) images displaying the height characteristics of graphene films transferred with the assistance of PMMA and e) paraffin support layers. f) Raman spectrum of graphene transferred on to a Si/SiO_2_ substrate utilizing both PMMA and paraffin methodologies. g) Diagrams illustrating how paraffin’s thermal expansion impacts the formation of wrinkles in graphene. a-e) Reprinted with permission under a Creative Commons (CC BY 4.0) License from ref (56). Copyright 2019 Nature Publishing Group.

While it cannot be denied that PMMA is an easily accessible polymer for transferring 2D films, the presence of cracks and PMMA residues cannot be overlooked if the PMMA removal process is not meticulously and comprehensively executed. These issues can significantly impact material quality and hinder achieving optimal performance when integrated into devices.^88−91^ In a report by Choi et al., it was pointed out that organic residues and cracks that occur after PMMA removal are the primary factors contributing to increased electrical resistance, ultimately leading to a decline in device performance.^92^ Exploring alternative polymers was the initial approach to mitigate reliance on PMMA, which could pose certain limitations during experimental procedures. Paraffin has been demonstrated to provide robust support and enhance the 2D film transfer process among various polymers. In this regard, Leong et al. employed paraffin as a supporting layer applied to graphene on a Cu foil substrate synthesized via CVD (Figure 7a-c).^56^ A clear distinction emerges when comparing paraffin-transferred graphene to PMMA-transferred graphene, with the former exhibiting a significantly smoother surface (Figure 7d, e). This disparity becomes even more apparent when analyzing Raman spectra, where the characteristic D band peak, indicative of defects, was conspicuously absent in paraffin-transferred graphene, affirming its high quality (Figure 7f). Moreover, paraffin is renowned for its high thermal expansion coefficient, resulting in tensile strain applied to the graphene film beneath it (Figure 7g). This strain stretched and reduced wrinkles in the graphene film, transforming graphene into a transfer-supporting layer and a means to render the graphene surface even flatter.

##### 2.1.1.4 Polypropylene Carbonate (PPC) Transfer

**Figure 8:**
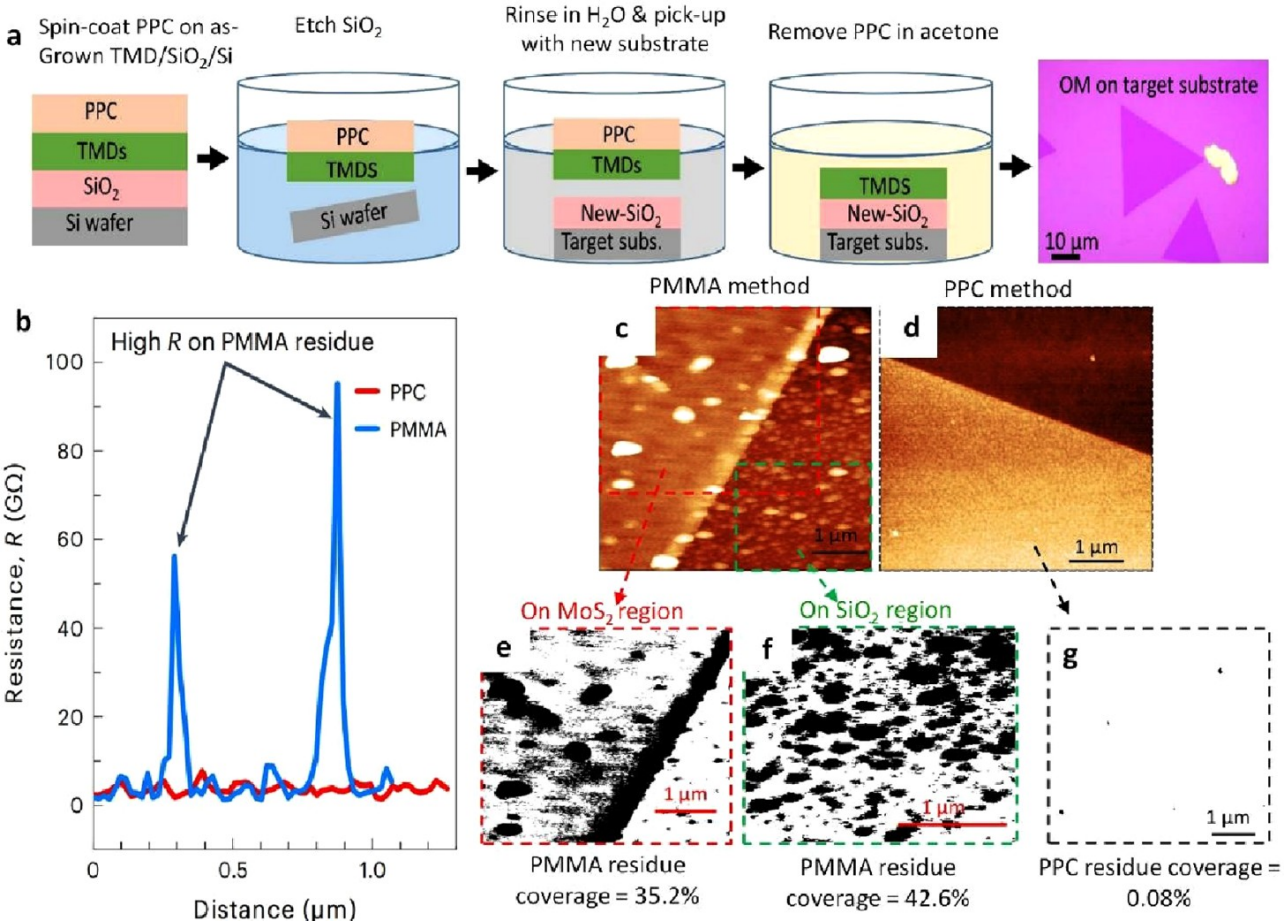
PPC-assisted transfer of MoS_2_. a) Schematic illustrates the transfer steps of MoS_2_ used by wet transfer and PPC. b) The resistance (R) peak of MoS_2_ was transferred using the PMMA and PPC method. c, d) AFM images illustrating the transfer of monolayer MoS_2_ on SiO_2_ substrate using conventional PMMA and PPC methods, respectively. e, f) Contrast images depicting PMMA residues (black spots) extracted from the AFM micrograph captured on MoS_2_ (enclosed by the red dashed box in e) and SiO_2_ (enclosed by the green dashed box in f) regions to assess residue coverage. g) PPC residue coverage was extracted from d). a-g) Reproduced with permission from ref (69). Copyright 2023 Nature Publishing Group.

In the most recent breakthrough in 2023, Modal et al. opted for PPC as the best candidate for the transfer technology of 2D film materials. This PPC transfer proved dominant with an ultraclean compared with the other transfer technologies (PMMA, PDMS, etc.).^69^ Visual inspection of optical micrographs readily reveals a substantial presence of PMMA residues on both the MoS_2_ surface and substrate (Figure 8a). Conversely, the final product is nearly pristine with PPC transfer, leaving behind no discernible residues or surface defects. This assertion is corroborated by measuring the electrical resistance of MoS_2_ samples after transfer using PMMA and PPC. The average resistance (R_mean_ = 1.5 GΩ) of the sample transferred using PPC is significantly less than the R_mean_ of 4.6 GΩ observed in the sample transferred using PMMA. This substantiates that PPC-transferred MoS_2_ exhibits a more uniform surface. In contrast, PMMA-transferred MoS_2_ exhibits several points with resistance soaring to 95 GΩ, attributed to the presence of PMMA residues (Figure 8b). In greater detail, an in-depth analysis of the AFM images was conducted to accurately measure the extent of residue coverage, as illustrated in Figure 8c-g, demonstrating a negligible PPC residue coverage of −0.08 ± 0.0065%. This starkly contrasts with PMMA residues, exhibiting a −35 ± 0.0059% coverage on MoS_2_. A similar pattern was noted on the SiO_2_ substrate, where PPC residue was nearly nonexistent when contrasted with PMMA (−043 ± 0.0061% PMMA coverage).

#### 2.1.2 Electrochemical-Assisted Transfer

**Figure 9:**
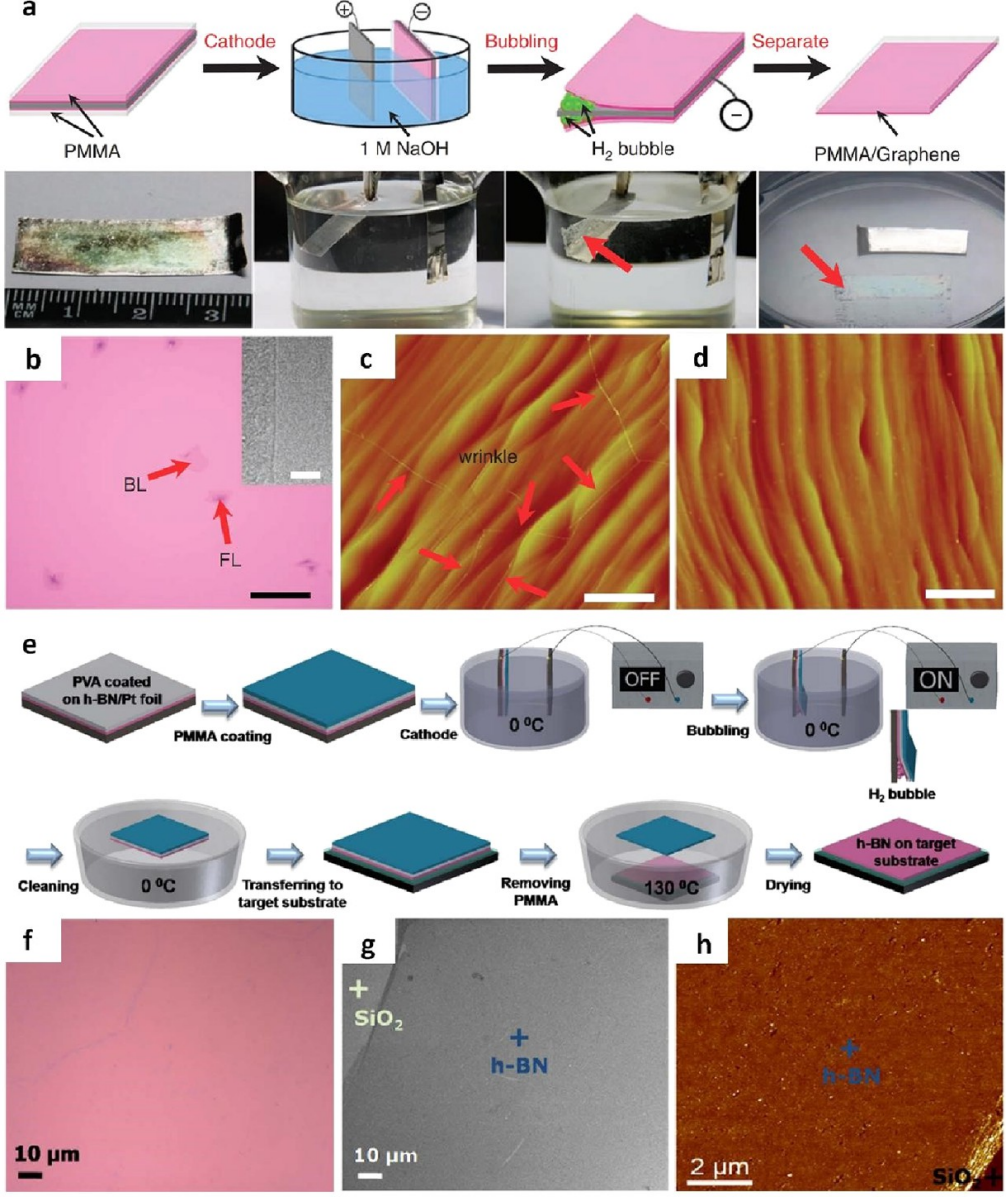
Electrochemical-assisted transfer of graphene and h-BN. a) Depiction of the electrochemical transfer process for graphene from a Pt substrate. b) Typical visual representation of the graphene transfers onto a SiO_2_/Si substrate, revealing predominantly monolayer graphene with some bilayer and few-layer areas. The inset highlights the edge of a monolayer graphene. The scale bar is set at 10 μm. c) AFM images of the Pt (111) surface postgraphene growth and d) subsequent bubbling transfer, indicating the Pt (111) surface maintains its original structure after the transfer. The wrinkles observed in c) confirm the presence of graphene. The scale bars in c) and d) are 2 μm. a-d) Reproduced with permission from ref (94). Copyright 2012 Nature Publishing Group. e) Schematic of electrochemical-assisted transfer with a support layer of PVA/PMMA for single layer h-BN. f) Bright-field optical microscopy image, g) SEM image, and h) AFM images illustrating a single layer of h-BN on SiO_2_/Si substrate, transferred using the electrochemical assisted transfer method with PVA/PMMA. e-h) Reprinted with permission under a Creative Commons (CC BY 4.0) License from ref (95). Copyright 2016 Nature Publishing Group.

Although polymer-assisted transfer stands out as an accessible method with a relatively straightforward execution process, it has inherent limitations, particularly in the context of removing the growth substrate to obtain a polymer/2D film membrane. This step is nearly nonintervenable, and reactions between the samples and the etching solution occur naturally. This is also why several methods have emerged to enhance and provide deeper insights into the transfer process of 2D films. Among them, electrochemical-assisted transfer is a promising approach that delivers positive results.^93^ In essence, by applying electrical potential or current, materials can be delicately and precisely separated, enabling a controlled transfer onto a target substrate. In Gao et al.’s report, a PMMA/graphene/Pt composite was immersed in a sodium hydroxide (NaOH) aqueous solution and utilized as the cathode in an electrolysis cell supplied with a constant current (Figure 9a).^94^ However, when employing PMMA/graphene/Pt as the cathode, graphene may be undergoing oxidation. This is attributed to the positive charges at the anode, where a water solution oxidation reaction generates O_2_, while at the anode, a water reduction reduction reaction yields H_2_. The results have unequivocally demonstrated that electrochemical-assisted transfer is a process that preserves the structural integrity of graphene (Figure 9b). Furthermore, it is worth emphasizing that atomic terraces are meticulously maintained, with no degradation in surface roughness, even after undergoing numerous growth and transfer cycles (Figure 9c, d). Importantly, this method leaves no traces of graphene fragments or wrinkles. In another improvement, Ngoc et al. employed this technique to transfer h-BN, incorporating a PVA buffer layer between h-BN and PMMA (Figure 9e).^95^ This strategic addition serves to minimize PMMA residue during the cleaning step involving hot water, effectively reducing contamination to a minimum.

The quality of h-BN has been thoroughly validated and confirmed through optical microscopy, SEM, and AFM, all of which have revealed a pristine surface completely devoid of any contamination (Figure 9f-h). However, within the same articles, the authors also point out that graphene transferred using the electrochemical-assisted transfer with a support layer of PVA/PMMA can still exhibit some residual contamination on its surface. These impurities are confined at the boundary between the transferred graphene and the destination substrate, leading to a decline in its electrical characteristics and diminishing the dependability of devices reliant on graphene. In contrast, with a modification of the delamination process, Wang et al. and Yang et al. harnessed the interaction between the acid tetra-*n*-butylammonium acetate (CH_3_COOTBA) and phosphorus atoms to achieve larger domain sizes of a few layers of black phosphorus.^96,97^ Consequently, the electrochemical-assisted transfer becomes evident that it is an effective method, allowing for the complete reusability of the growth substrate. However, it is crucial to carefully consider the potential interactions that may occur during the detachment process to prevent undesirable effects and the presence of unintended reaction byproducts on the material’s surface before this procedure is executed.

### 2.2 Dry Transfer

Unlike wet transfer, which requires ionic liquids to detach the material from the growth substrate, the dry transfer method proves to be a simpler and more cost-effective approach, all while ensuring material quality.^98−101^ By eliminating ionic liquid during the material removal process from the initial substrate, this method mitigates the potential hazards of liquids, such as contamination and defects in the transferred material or the intended application. Therefore, dry transfer has become an increasingly intriguing technique within the scientific community. This section delves into recent advancements in transferring 2D films related to this method. Table 2 lists reports on the transfer and application of 2D films that used this method.

**Table 2:** Dry-Transfer Strategies on Representative 2D Films and Related Applications^a^

2D Films	Support layer	2D Films Size	Devices	ref
Graphene	PS	1 × 1.5 cm^2^	–	(102)
Graphene	Polyvinylpyrrolid one (PVP)/PVA Stamp	10 × 10 mm^2^	Si Solar cells	(103)
Graphene	Polyethylene terephthalate (PET)/Silicone stamp	1 × 1 cm^2^	Perovskite Solar cells	(104)
Graphene	PET/Silicone stamp	8 × 3 cm^2^	Quantum dots LEDs	(105)
Graphene	PDMS	–	FETs	(106)
Graphene	PET/PSA stamp	21 × 29.7 cm^2^	Micro- Super capacitors	(107)
Graphene	PMMA/PVA stamp	–	–	(108)
Graphene	PMMA	76.2 cm	Flexible FETs	(109)
Graphene	Adhesive- strained layer (Ni)	10 cm	FETs	(110)
WS_2_	PDMS	–	FETs	(111)
WS_2_ and ReS_2_	PDMS	–	–	(112)
WS_2_	Au- and Ag- assisted transfer	–	LED	(113)
WS_2_	Poly(bisphenol A carbonate)	10 × 10 μm^2^	–	(114)
WS_2_	PDMS	–	Perovskite Solar cells	(115)
MoS_2_	PDMS	–	–	(116)
MoS_2_	PC film	–	Light-induced energy applications	(117)
MoS_2_	PVA/PMMA/PDMS stamp	–	Back-gate transistors	(118)
MoS_2_	Adhesive matrix of Au or Polymer	–	Transistors	(119)
MoS_2_ and GaSe	Nitto tape/PDMS	–	NO_2_ Sensing	(120)
MoS_2_	PC/PDMS stamp	–	Straintronics	(121)
MoS_2_	Au/PS stamp	–	Electro catalytic	(122)
MoS_2_	PDMS	–	Mechanical Resonators	(123)
MoS_2_ and GaTe	PDMS	–	Sensitive Photodetector	(124)
MoS_2_	PDMS	–	FETs	(125)
h-BN	PMMA/Polymethylglutar imide (PMGI)	2 × 2 cm^2^	–	(126)
h-BN	PDMS	–	Heater and Sensor	(127)
Graphene, ReS_2_, and h-BN	PPC	–	Tunneling Diodes, FETs, Logic gates, and Memory	(128)
Graphene, and h-BN	Au-assisted transfer	–	–	(129)
Tantalum sulfide (TaS_2_), and MoS_2_	PDMS	–	FETs	(130)
NbSe_2_, and Graphene	Poly(4styrenseulfonic acid) (PSS)	–	FETs	(131)
BP	Poly(vinyl chloride) (PVC)	–	FETs	(132)
BP	PDMS	–	Nanoelectroche mcal resonators vibrating	(133)
BP	Gel-Pak (gelfilm)	–	Gas Sensors	(134)

a Here, “–” means “not applicable”.

#### 2.2.1 Dry Transfer via Polymer Supporting Layer

##### 2.2.1.1 PDMS Transfer

**Figure 10:**
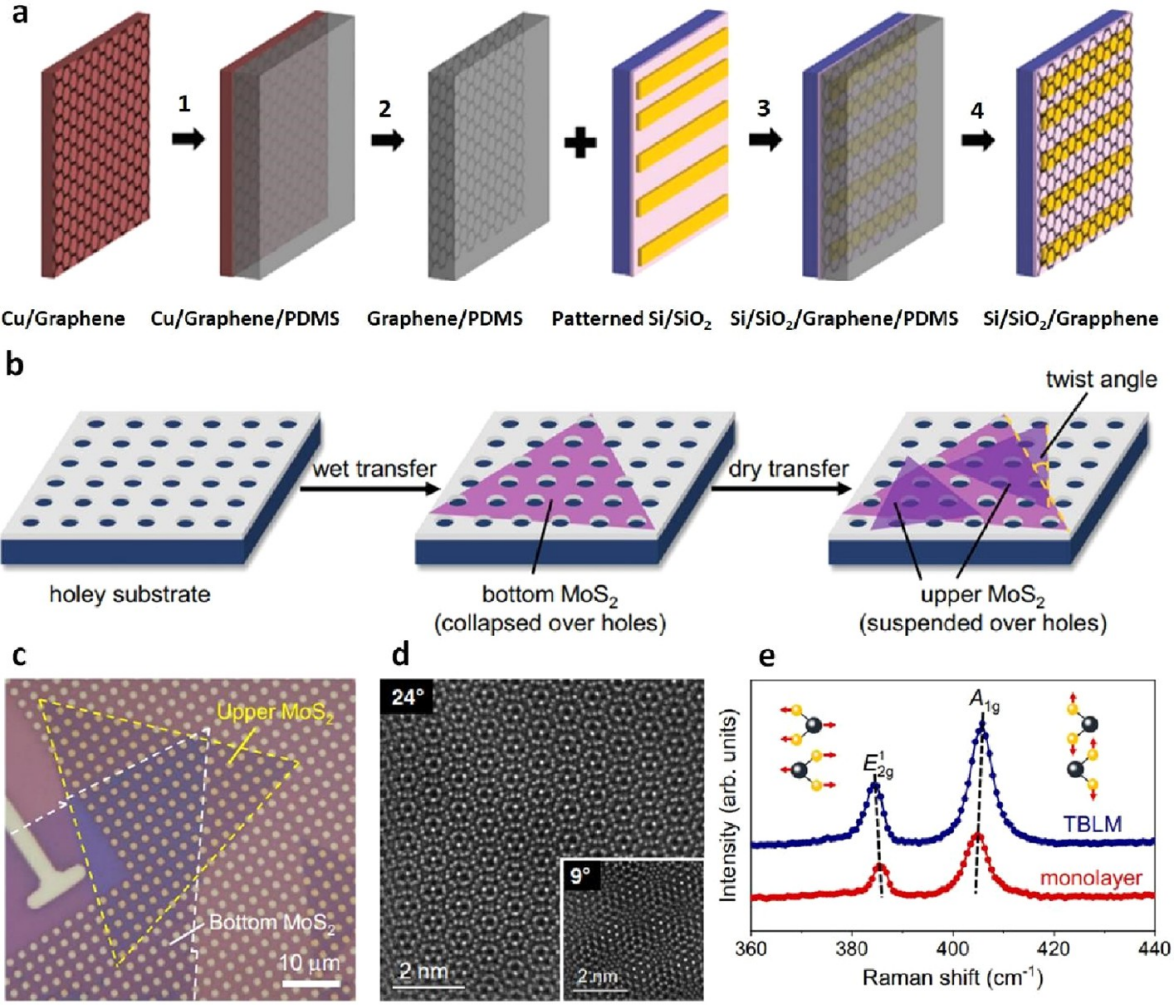
PDMS-assisted dry transfer of graphene and MoS_2_. a) Schematic depiction of the CVD graphene transfer process: (1) adhering Cu/graphene to PDMS; (2) Cu etching; (3) affixing graphene/PDMS to Si/SiO_2_; (4) removing PDMS. Reproduced with permission from ref (106). Copyright 2015 Royal Society of Chemistry. b) Illustration depicting the procedure for creating a twisted bilayer MoS_2_. In this scenario, the lower layer of MoS_2_ folds over the perforations while the upper layer is suspended above them. c) Visual representation of a sample of twisted bilayer MoS_2_. The dashed lines outline the boundaries of the upper and lower MoS_2_ monolayers. d) Cler Moiré patterns of twisted bilayer MoS_2_ at twist angles of 24° and 9° (inset) observed through annular dark-field scanning transmission electron microscopy (STEM). e) A comparative analysis of the Raman spectra of twisted bilayer MoS_2_ and monolayer MoS_2_. b-e) Reprinted with permission under a Creative Commons (CC BY 4.0) License from ref (116). Copyright 2022 Nature Publishing Group.

In the early studies related to the utilization of PDMS, Meitl et al. demonstrated the exceptional potential of PDMS as an elastomeric stamp for material transfer when the separation energy (G_PDMS_) at the elastomer-material interface is sufficiently robust to overcome the material-substrate interface through an accelerated delamination process.^135^ In 2015, Abhilash et al. employed a PDMS method coated onto graphene to effectuate the transfer from a Cu substrate to a Si/SiO_2_ substrate (Figure 10a).^106^ The graphene surface was nearly flat, with a roughness of approximately ±3 nm. The authors also acknowledge the possibility of rips and wrinkles arising during the Cu/graphene/PDMS assembly preparation phase. In a scenario involving the twisted bilayer MoS_2_, dry transfer using PDMS significantly aided in stacking and suspending the upper MoS_2_ monolayer region over a hold (Figure 10b).^116^ After the dry transfer process, the upper layers of MoS_2_ were randomly arranged on top of the lower layers, creating a bilayer MoS_2_ with unpredictable twist angles (Figure 10c, e).

However, in some cases, the direct use of PDMS and 2D films may inadvertently impact material quality. This is especially critical in applications related to the formation of 2D heterostructures because the adhesion strength of PDMS varies for different 2D films. For example, the adhesion of PDMS to graphene is weaker than PDMS to h-BN. Consequently, structural distortions within this heterostructure may occur during the dry transfer process from PDMS/graphene to h-BN. Additionally, the surface of PDMS tends to be rougher than that of PMMA or other polymers. Thus, finding a solution that harnesses the strengths of PDMS without compromising the desired structures is of utmost importance.

##### 2.2.1.2 Integrated PDMS/PPC Transfer

**Figure 11:**
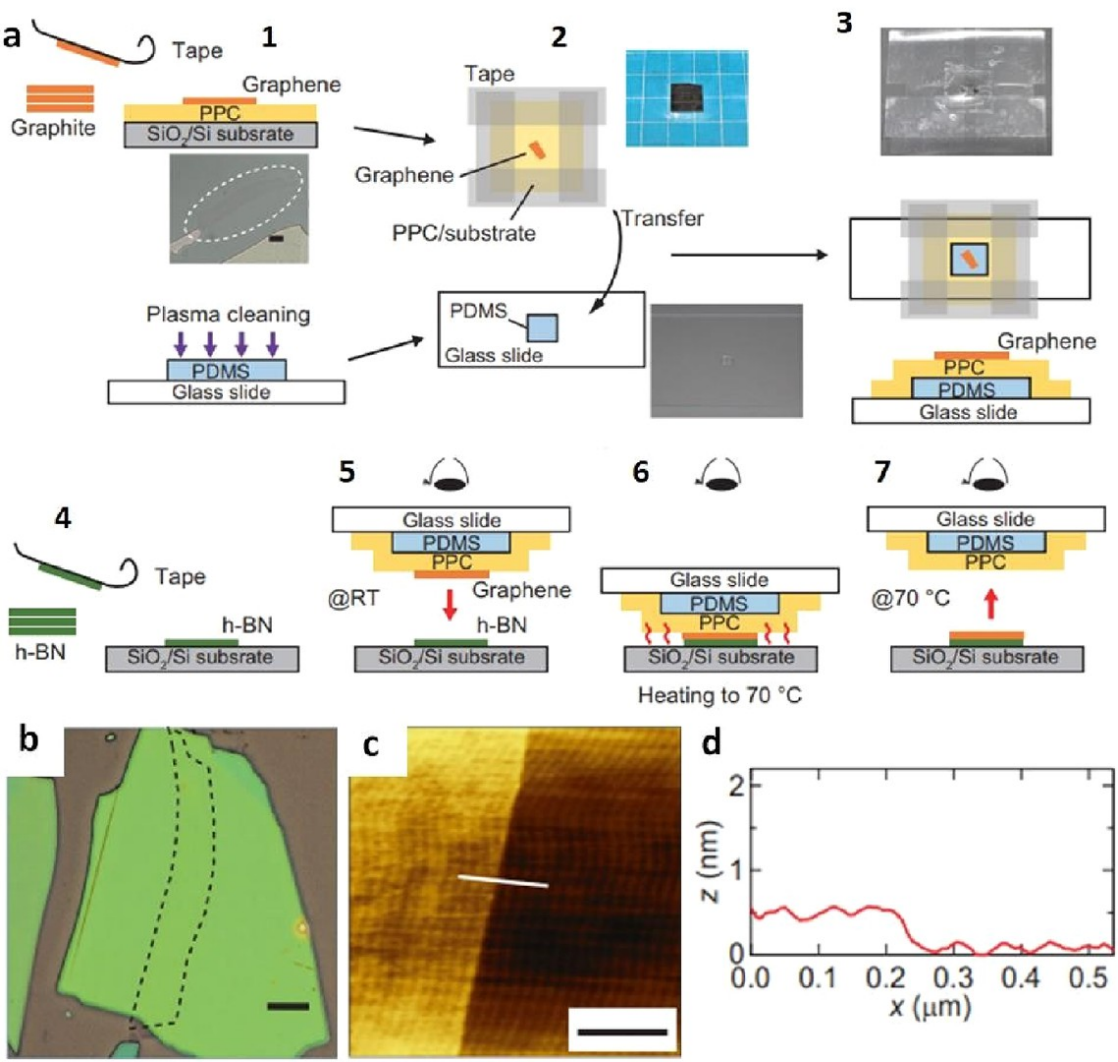
Combined PDMS and PPC assisted dry transfer process for graphene on h-BN/SiO_2_/Si substrate. a) Illustrations outlining the process of the dry transfer method, including (1) the preparation of graphene on a PPC/SiO_2_/Si substrate; (2) the creation of a taped window surrounding the graphene area; (3) assembly of the prepared graphene/PPC/PDMS structure on a glass slide; (4) Employing mechanical exfoliation to obtain an h-BN flake with thickness of approximately 30 nm on SiO_2_/Si substrate; (5) aligning the positions of graphene and h-BN flakes and establishing a gentle contact at room temperature; (6) elevating the stage temperature to 70 °C while maintaining contact between graphene and h-BN. (7) delicately separating the glass slide from the SiO_2_/Si substrate. b) Optical micro images, (c) AFM topographic image, and (d) AFM height profile of single layer graphene on thick h-BN. a-d) Reprinted with permission under a Creative Commons (CC BY 4.0) License from ref (136). Copyright 2019 Nature Publishing Group.

In another approach, the effectiveness of 2D film exfoliation can be significantly improved. Combining PDMS with other polymers can create robust bonds with the material during the transfer process, making it more straightforward and yielding superior results. Graphene provides an exemplary case for such transfers, employing a combination of PMMA and PDMS. Here, it functions as an adhesive and safeguards the materials, while PDMS acts as an exfoliation to remove PMMA. In the study by Tien et al., graphene/PMMA/PDMS was meticulously aligned with an accuracy of a few microns on the target h-BN flake.^108^ Subsequently, PDMS and PMMA were retracted by heating to approximately 90 °C for about 10 min. The result was the creation of a 2D heterostructure comprising graphene and h-BN via an all-dry transfer process. In a similar application involving a graphene-hBN heterostructure, Kinoshita et al. opted for PPC as a substitute for PMMA (Figure 11a).^136^ As discussed in section 2.1.1, PPC exhibits strong adhesion to 2D films at room temperature, which significantly diminishes at higher temperatures. Additionally, removing residual PPC from the graphene surface only requires annealing at approximately 350 °C, eliminating the need for graphene or h-BN to be exposed to organic solvents throughout the process. The results further confirm that the surface of monolayer graphene on a thick h-BN/SiO_2_/Si heterostructure remains free from noticeable polymer residue (Figure 11b-d). Hence, the PDMS-assisted dry transfer method based on PPC emerges as an exceptionally promising and suitable approach for fabricating 2D heterostructures.

#### 2.2.2 Thermal Release Tape-Assisted Transfer

**Figure 12:**
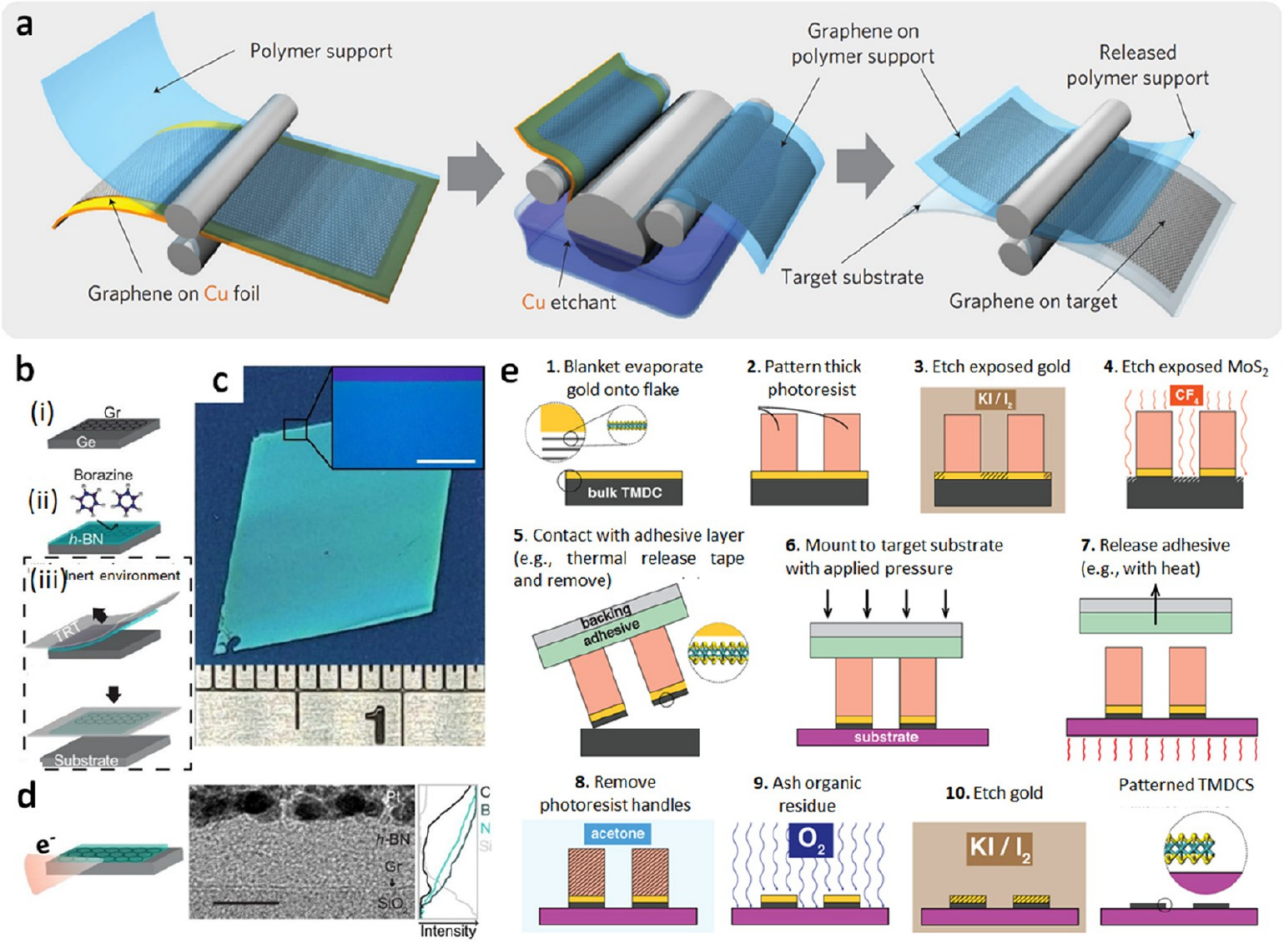
TRT-assisted dry transfer of graphene, hBN, WS_2_ and MoS_2_. a) Diagram illustrating graphene film production grown on a Cu foil through a roll-based process. Reproduced with permission from ref (109). Copyright 2010 Nature Publishing Group. b) Visual representations outlining the all-dry transfer process of a graphene film using van der Waals interactions: (i) initiating graphene growth on Ge (110), (ii) promoting h-BN growth, (iii) mechanically exfoliating the h-BN/graphene hybrid film via TRT, and (iv) transferring the film onto diverse substrates. c) Illustration depicting the successful transfer of a 50 nm h-BN/graphene film onto a SiO_2_/Si substrate with a scale bar of 100 μm. d) (left) Schematics illustrations portraying cross-sectional TEM, (middle) TEM image, and (right) EDX intensity profile. Scale bar: 10 nm b-d) Reproduced with permission from ref (138). Copyright 2019 American Chemical Society. e) Process for producing patterned monolayer throughout TRT. Reproduced with permission from ref (140). Copyright 2019 American Chemical Society.

Another method aimed at enhancing and minimizing the impact of organic residues on the material’s surface has been under development. This method is based on the thermal decomposition of organic residues. The thermal release tape (TRT) plays a pivotal role in this approach, as it is designed to respond to heat.^137^ When subjected to heat, this specialized tape releases, facilitating the transfer of 2D films onto the desired substrate. The initial advancements in this technique were pioneered by Bea et al. in 2010.^109^ In their groundbreaking work, they successfully transferred a graphene film from TRT onto a Polyethylene Terephthalate (PET) film in a roll-to-roll process at 120 °C. Since then, the utilization of TRT in material transfer has garnered significant attention, emerging as a practical and optimized solution for transferring 2D films (Figure 12a).

In this manner, Yang et al. successfully executed the transfer of a graphene/h-BN heterostructure.^138^ After growing and h-BN on germanium (Ge) (110) substrates through CVD. They applied a PMMA layer as a TRT onto the samples. The TRT was subsequently released by heating the substrate to 135 °C and PMMA would be removed at 350 °C on hot plate (Figure 12b). Furthermore, to determine the composition of the transferred film, an energy-dispersive X-ray (EDX) elemental mapping was carried out by TEM (Figure 12c, d). Along the interfaces, the EDX intensity profiles for each element, such as carbon, boron, nitrogen, and silicon, clearly delineated the presence of both h-BN and graphene layers on the SiO_2_ substrate. On the other hand, Lin et al. employed a PDMS layer as a TRT for the dry transfer of molybdenum diselenide (MoSe_2_) to a Si substrate.^139^ In this process, the substrate was heated to 105 °C for 2 min and then allowed to cool to room temperature, after which the TRT was carefully removed. This technique is acknowledged for its capability to directly transfer patterned monolayer WS_2_ and MoS_2_, featuring sizes exceeding 10^4^ μm^2^, from multilayer sources.^140^ Following exfoliation from the source, the material transfers through TRT onto SiO_2_ substrate, where heat and pressure are applied. Subsequently, the tape and photoresist are removed, and the remaining Au is subjected to etching (Figure 12e). It can be said that this is a valuable approach for transferring materials over a large area, making it amenable to easy intervention and control of conditions during the transfer process. However, attention to detail is essential, particularly concerning materials sensitive to temperature, heating times, and the thickness of TRT, to achieve the best possible results.

#### 2.2.3 Ultraviolet (UV) Light-Assisted Transfer

**Figure 13:**
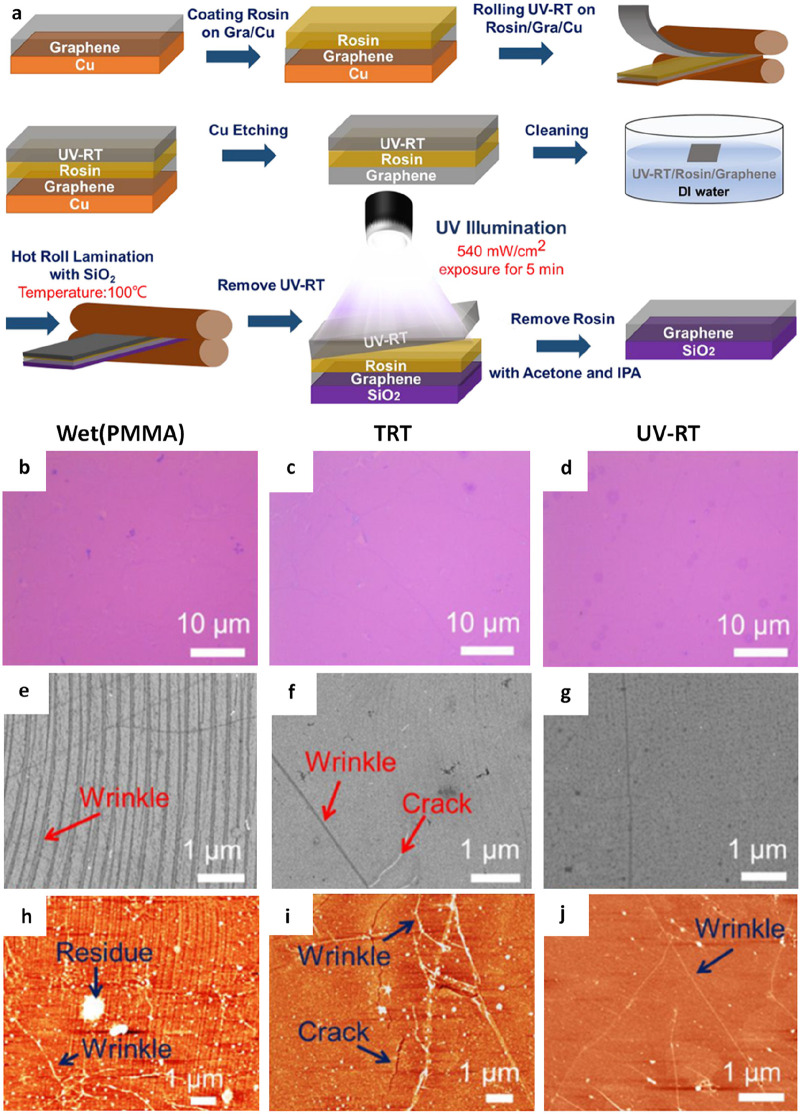
UV-light-assisted dry transfer of graphene. a) Schematic depiction of the UV-RT transfer procedure. b-d) Concomitant optical microscopy images, e-g) Scanning electron microscopy (SEM) images, and h-k) AFM images depict the morphological characteristics of graphene transferred through wet transfer, TRT transfer, and UV-RT transfer methods. a-g) Reproduced with permission from ref (142). Copyright 2023 American Chemical Society.

The application of TRT has facilitated the large-area transfer of 2D films. Nevertheless, these procedures and mechanisms require meticulous management of temperature or pressure to avoid uneven polymer detachment caused by the inherent variations in heat and pressure distribution.^141^ Addressing this challenge, a recent report by Hung et al. explored the use of UV light as a tool to support dry-transfer processes for graphene.^142^ In this research, an efficient transfer of graphene film was accomplished using a roll-to-roll (R2R) continuous system in conjunction with an innovative UV release tape (UV-RT), ensuring swift and effective transfer. Initially, a layer of Rosin was coated onto graphene/Cu, followed by the UV-RT being rolled onto these layers. Afterward, conventional wet transfer was employed to eliminate the Cu, producing a composite film comprising UV-RT, rosin, and graphene. Utilizing an R2R process, this composite film was affixed to the desired substrate, and the rapid release of UV-RT was achieved through exposure to UV light. Ultimately, the polymer was extracted, leaving the graphene securely on the target substrate (Figure 13a). Additionally, a comparison among wet-transfer PMMA, TRT, and UV methods was conducted, revealing that wet-transferred graphene exhibited high roughness and difficulty in removing polymer residue. On the contrary, the graphene transferred via UV-RT using dry-transfer exhibited fewer cracks and residues, resulting in a cleaner surface when compared to graphene transferred via TRT. The diminished damage to graphene during UV-assisted dry transfer was attributed to the TRT detachment mechanisms, which involve foaming and molecular stripping after heating, leading to uneven stress distribution (Figure 13b-j). On the flip side, the UV-RT detachment mechanism, triggered by UV light exposure, swiftly and uniformly transformed its molecular phase into a three-dimensional network structure. This process minimized adhesion strength and mitigated uneven stress, consequently causing less harm to the graphene. In summary, UV-assisted dry transfer is a technique capable of swiftly transferring materials onto target substrates in one step. UV light plays a dual role, releasing the UV tape and deteriorating the adhesion layer into easily removable molecules, achieving an ultraclean material surface with high cleanliness.

#### 2.2.4 Metal-Assisted Dry Transfer

In another context, Silicon carbide (SiC) and sapphire substrates are renowned as ideal platforms for forming 2D films.^141−147,143−149^ A prime example is the growth of graphene on SiC substrates, which has been extensively researched and demonstrated to result from the sublimation of silicon at high temperatures, leaving a carbon-rich surface on the SiC substrate and rendering it an ideal environment for graphene growth.^150,151^ However, this direct growth process leads to strong bonding between graphene and the SiC surface. Therefore, in transfer applications, a material such as Nickel (Ni), Gold (Au), or Palladium (Pd), with superior bonding capability, is required to facilitate the step-by-step exfoliation of 2D film layers.

**Figure 14:**
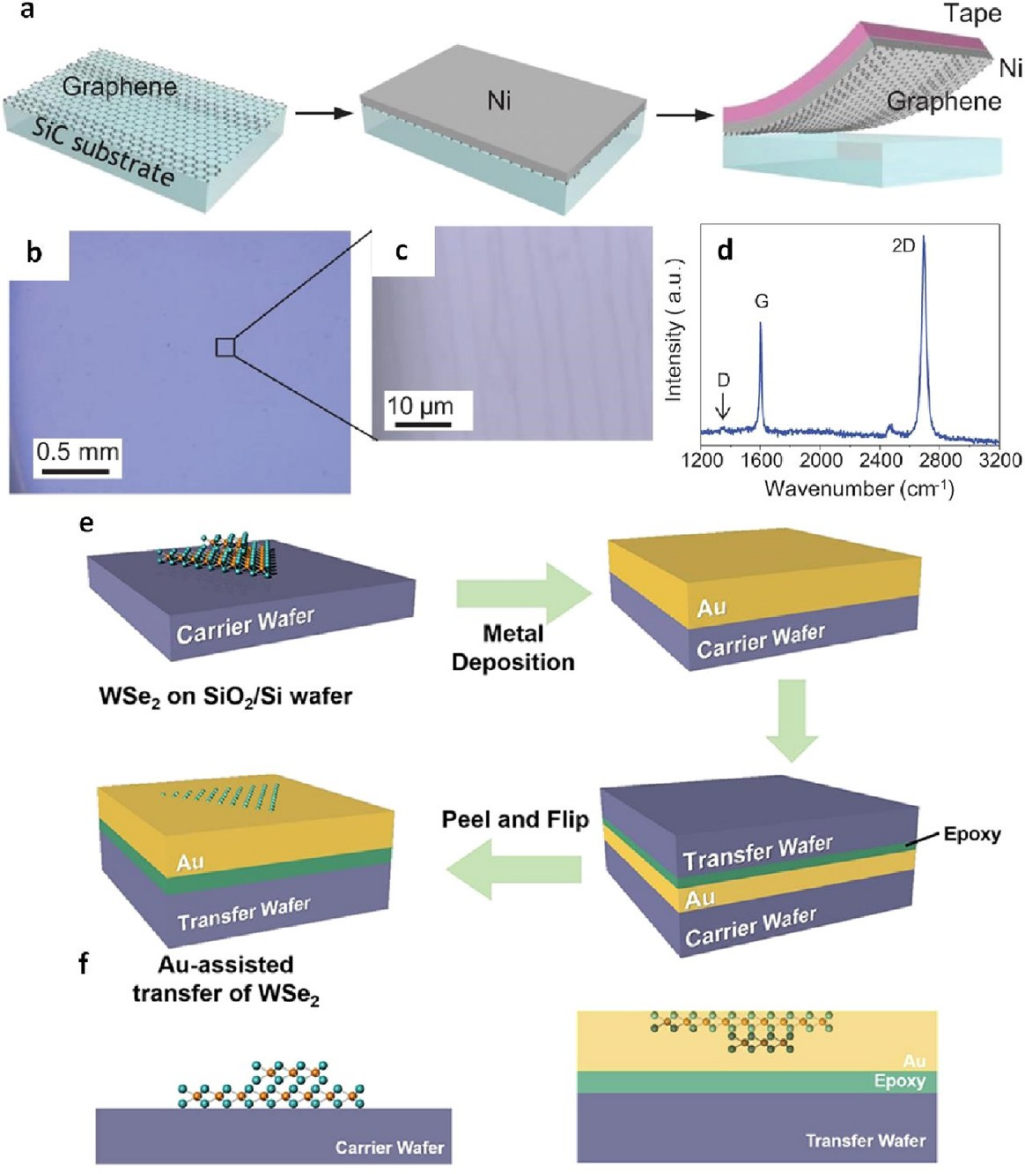
Metal-assisted dry transfer for graphene and WSe_2_. a) Illustration of the procedure to directly transfer graphene from a SiC surface onto SiO_2_/Si wafer. b) and c) Optical microscope snapshots of the graphene obtained from a recycled SiC wafer, demonstrating a highly successful transfer yield. d) Exemplary Raman spectrum of the graphene obtained from a reused SiC wafer, affirming the single-layer nature and the undamaged state of the transferred graphene (absence of D peak) a-d) Reproduced with permission from ref (110). Copyright 2013 AAAS. e) Sequential diagram illustrating the Au-assisted transfer process of CVD-grown WSe_2_ crystal, employing thermal epoxy as the bonding layer. f) Cross-sectional perspective of the samples both before and after transfer. e, f) Reproduced with permission from ref (152). Copyright 2019 American Chemical Society.

Kim et al.’s report vividly showcases the intricate steps in achieving a consistent and replicable process for exfoliating and transferring epitaxial graphene directly from a SiC substrate onto a different substrate.^110^ To accomplish this, the authors ingeniously employed an adhesive-strained layer of Ni and a thermal release tape to peel the graphene from the SiC substrate delicately (Figure 14a). Their calculations revealed the binding energy per atom between graphene and Ni (γ _Ni-graphene_) to be approximately 140 meV, a testament to the significant ability of Ni to undergo this high-strain transfer. Subsequent experiments confirmed that the quality of the transferred graphene remained uncompromised throughout the process, thanks to the utilization of the Ni layer (Figure 14b, c). Raman measurements further substantiated this, as they exhibited no presence of the D peak in the spectra, signifying that the quality of graphene remained untarnished by the Ni deposition layer (Figure 14d). In addition to Ni, Au also emerges as a prominent choice for assisting in transferring materials like WSe_2_ or MoS_2_.^152^ In this scenario, thin layers of Au are typically deposited onto the material using a thermal evaporation method. After evaporation, a piece of Si wafer is adhesively attached to the noble metal film using a fine layer of thermal epoxy. Once the epoxy layer has fully cured and achieved optimal hardness, the upper Si piece is gently peeled upward, effectively stripping the Au film and the WSe_2_ crystal from the substrate (Figure 14e, f). Overall, this method underscores the exceptional capability for high-quality transfers. However, it is imperative to consider crucial factors before the transfer, such as ensuring the bond between the metal layer and the 2D films is sufficiently robust and exceeds the bond between the materials and the substrates. Furthermore, the cost and the metal deposition process can pose significant challenges in achieving a consistent thickness during the transfer process.

#### 2.2.5 Adhesive Matrix-Assisted Transfer

**Figure 15:**
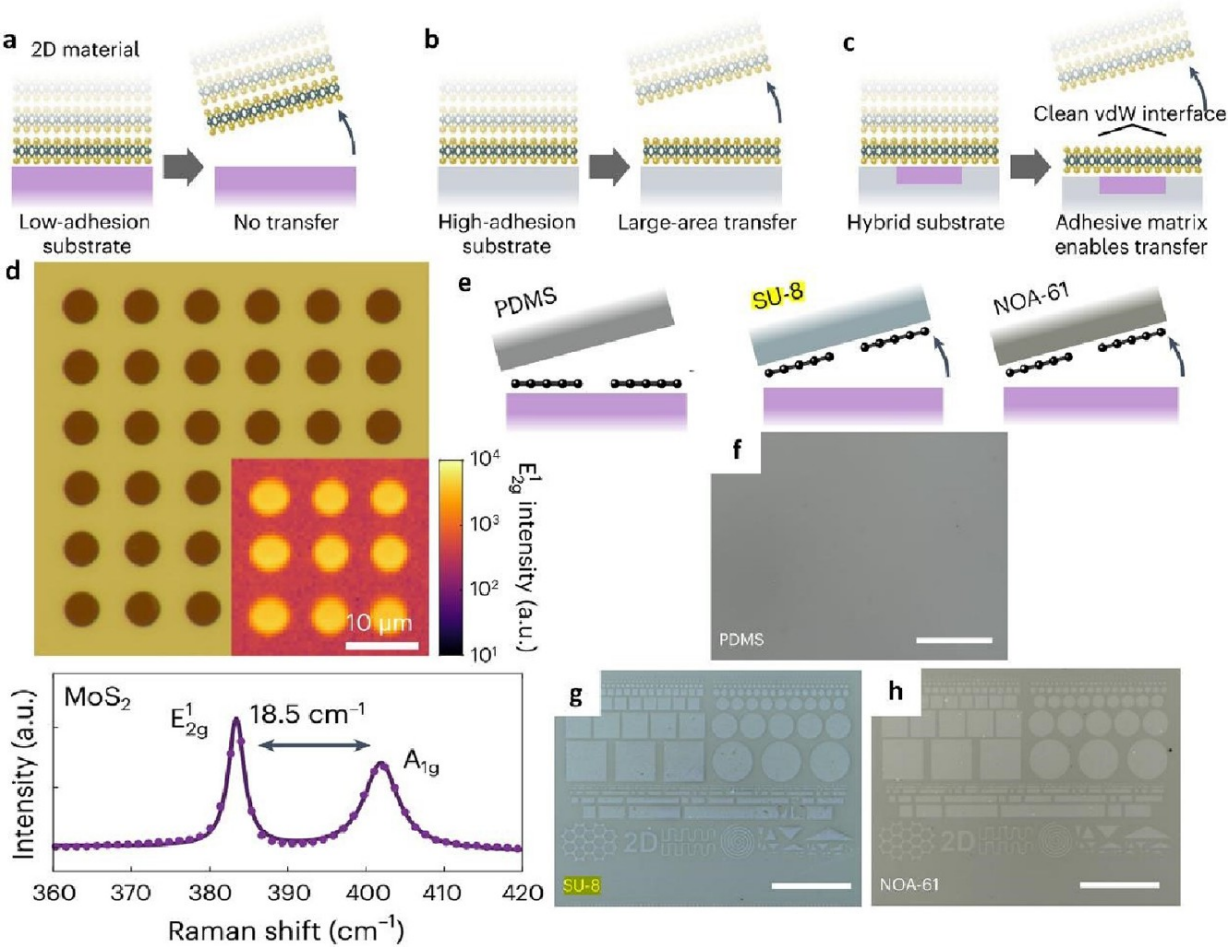
Adhesive matrix-assisted transfer of MoS_2_ and graphene. a) Schematic depiction of adhesive matrix transfer: lack of transfer is observed when a 2D film contacts a low-adhesion substrate; b) achieving large-area transfer of a continuous monolayer becomes feasible with specific high-adhesion substrate; c) embedding the low-adhesion substrate within a matrix of high-adhesion material facilitates the direct fabrication of clean van der Waals interfaces. d) By incorporating low-adhesion SiO_2_ into a gold matrix with adhesive properties that can directly craft MoS_2_/SiO_2_ heterostructures, a fact supported by Raman spectroscopy observations. e) Experiment for determining the appropriate adhesive matrix for patterned CVD graphene. Patterned graphene on SiO_2_ is prepared and connected with different templates’ stripped polymers. No transfer is observed to PDMS (f), and a high-yield transfer is observed to both SU-8 (g) and NOA-61 (h). Van Der Waals Device Integration beyond the Limits of van Der Waals Forces Using Adhesive Matrix Transfer. a-h) Reproduced with permission from ref (119). Copyright 2023 Nature Publishing Group.

Despite the dramatically achievements of current transfer methods in facilitating the high-quality transfer of 2D films, they still harbor the potential for damage and contaminants due to the removal of support layers. This poses challenges in integrating them into semiconductor devices to achieve optimal efficiency. An innovative approach to addressing this issue involves transferring 2D films to devices without the need for sacrificial layers, solvents, high temperatures, or post-transfer fabrication. This innovative dry transfer method is scalable and allows for precise alignment -a feature lacking in existing approaches. A delicate balance of surface interactions is crucial to succeed in this contact-and-release transfer. Higher adhesion is required at the 2D films/receiving substrate interface than at the 2D films/source interface. However, relying solely on van der Waals interactions proves insufficient for arbitrary heterostructures since these forces cannot be tailored on demand. In a recent report by Satterthwaite et al., an adhesive matrix transfer was employed as an important factor in order to achieve monolayer MoS_2_.^119^ This involves immersing a low-adhesion substrate into a matrix that can facilitate the transfer through strong adhesive interactions with the 2D films (Figure 15a-c). For instance, a matrix of Au was chosen for its ability to form robust adhesive interactions with MoS_2_, the material being transferred. When the hybrid substrate contacts the 2D films, the adhesive interactions with the matrix promote a successful transfer. This approach enables widespread van der Waals integration by overcoming the limitations of van der Waals forces. Optical microscopy images and Raman spectra confirm the successful transfer of continuous monolayer MoS_2_ from its source crystal onto an Au substrate embedded in SiO_2_ (Figure 15d). The observation of an 18.5 cm-1 separation between the E^1^_2g_ and A_1g_ Raman modes validates the presence of a continuous monolayer MoS_2_-on-SiO_2_. Importantly, this transfer method ensures the pristine state of the monolayer MoS_2_, as no polymer support layers or solvents were used throughout the process.

In the same report, the authors demonstrate that creating patterns on graphene can be effortlessly achieved by employing adhesive polymer matrices. Specifically, in the quest to identify a suitable polymeric adhesive matrix for graphene, a comparative study involving template-stripped PDMS, SU-8, and NOA-61 was conducted (Figure 15e). To ensure precise contact, the candidate matrices were brought into contact with patterned graphene and gently heated to 65 °C in a nanoimprint tool. Interestingly, no observable transfer occurred with PDMS, whereas high-yield transfers were observed for SU-8 and NOA-61 (Figure 15f-h). These matrices possess chemical composition and interaction that extend beyond van der Waals forces. This underscores the potential of adhesive matrix transfer, as separating the van der Waals heterostructure from the transfer matrix expands the range for engineering forces. This revelation presents an exciting opportunity for advancing force engineering in this context.

This emerging technique has garnered significant attention and interest owing to its convenient and superior features. The streamlined process minimizes transfer time and steps, rendering this method virtually impervious to external factors and ensuring the uniformity of 2D films throughout the transfer process. Consequently, this platform can be expanded, finding applications where clean, dry, and large-area van der Waals integration is crucial for scientific studies or device implementations.

#### 2.2.6 Inorganic Membrane-Assisted Transfer

In application involving the integration of two or more 2D films to form heterostructure, the role of transfer processes becomes increasingly crucial, particularly in controlling precision, finesse, and minimizing damage as well as contamination within each 2D film layer. Current approaches employing a polymer support to manipulate the van der Waals heterostructure are proving less effective in integration into semiconductor devices, where van der Waals heterostructure functions optimally only in contamination-free areas or under conditions of extreme cleanliness. Despite the excellent adhesion and flexibility of polymer layers, their residues persist at buried interfaces or on the surface of the completed heterostructure, depending on the method used.^153^ Consequently, more sophisticated strategies need to be researched and implemented for removing contamination from already assembled heterostructure.

**Figure 16:**
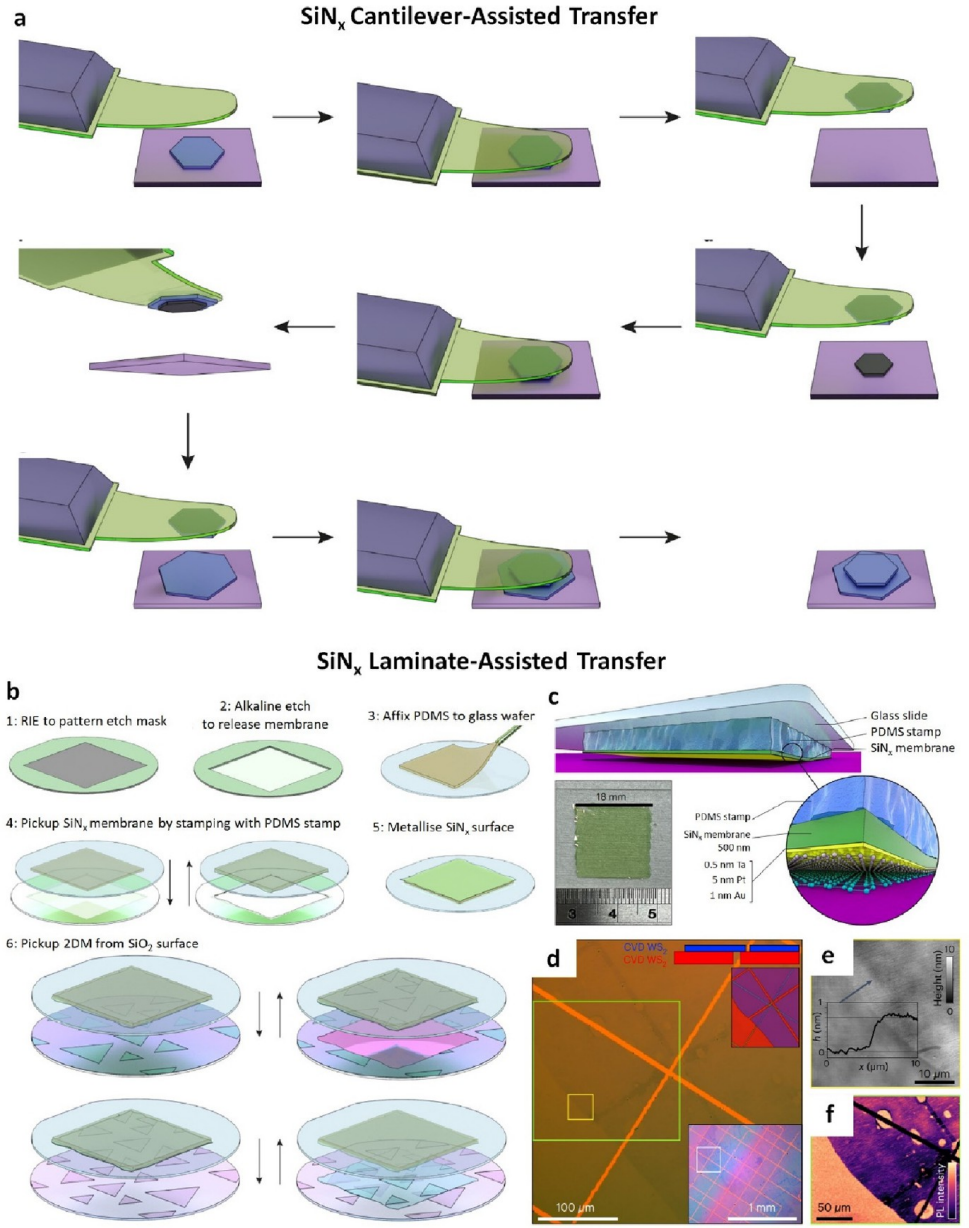
SiN_*x*_ membrane-assisted transfer of graphene, h-BN, MoS_2_, and WS_2_. a) Illustration of transfer method uses SiN_*x*_ cantilever for polymer-free heterostructure assembly. b) Schematic of the SiN_*x*_ laminate-assisted transfer. c) Schematic showing the arrangement of an SiN_*x*_ membrane supported by PDMS polymer. The inset shows an 18 nm SiN_*x*_ membrane laminated onto PDMS film. d) Heterostructure consisting of CVD-grown monolayer WS_2_ transferred onto CVD-grown few-layer WS_2_ on an SiO_2_ substrate fabricated using the laminates. The upper inset shows the two layers in red and blue to highlight the area covered. The lower inset shows a wider view with the location of the main image indicated by the white box. e) Topography of the area in d) indicated by the yellow rectangle. A height profile at the indicated position is shown in the inset, measuring a step of approximately 0.77 nm. f) Integrated intensity map of the primary WS_2_ photoluminescence peak around 1.97 eV. a-f) Reprinted with permission under a Creative Commons (CC BY 4.0) License from ref (154). Copyright 2023 Nature Publishing Group.

In a study by Wang et al., these challenges were addressed through a platform designed for fabricating pristine 2D heterostructures without the use of organic materials.^154^ An inorganic, flexible silicon nitride (SiN_*x*_) membrane coated with thin metallic films was utilized instead of a polymer layer in the transfer process. The transfer process relies on chemically inert, flexible, and transparent SiN_*x*_ membranes. However, the adhesion between the 2D film and SiN_*x*_ is relatively weak. To overcome this limitation, the authors applied a metal stack consisting of Ta, Pt, and Au. In which, Au known for its strong adhesion to 2D films, allows for tuning adhesion strength by adjusting its thickness for specific 2D/substrate combinations. The Pt layer compensates for variable SiN_*x*_ roughness, while Ta serves as the adhesion layer for Pt. The authors demonstrated high cleanliness achievable for the archetypal h-BN/graphene/h-BN vertically stacked heterostructure, fabricated from mechanically exfoliated 2D films on a Si/SiO_2_ substrate (Figure 16a). After successfully attaching a cantilever with h-BN, it is further utilized to pick up the graphene, and the resulting stack is deposited onto the bottom h-BN, leveraging greater adhesion to this larger bottom crystal. Similarly, the h-BN/MoS_2_/h-BN heterostructure exhibits no bubbles and has a completely clean 9 μm × 15 μm area where all eight layers’ overlap and also confirmed by overall AFM and local cross-section STEM measurements.

While cantilever geometry is a potential method for transferring CVD-grown materials, the inherent freestanding property of 500 nm-thick SiN_*x*_ poses challenges for scaling this technique beyond approximately 200 μm lateral scales. To address this issue, the authors developed a solution by laminating a metal-coated SiNx membrane onto a 0.432 mm-thick PDMS film (Figure 16b, c). Direct contact between the 2D films and the PDMS surface can lead to surface contamination from un-cross-linked oligomers within the PDMS bulk. However, the SiN_*x*_ membrane acts as an impermeable barrier against this contamination, allowing for the combination of the ultraclean interface of metallized SiN_*x*_ with the mechanical flexibility and support of PDMS. This innovative approach facilitated the transfer of a large CVD-grown WS_2_ sheet onto multilayer WS_2_ films grown on sapphire and SiO_2_ substrates, resulting in a bubble-free heterostructure with an AFM-measured step height of 0.7 nm (Figure 16d). These results indicate minimal contamination at the interface, consistent with a 50–60% reduction in photoluminescence intensity in the overlapping area (Figure 16e, f). Moreover, both the transfer technique and the SiN_*x*_ film itself are commercially available at wafer scale, offering superior chemical, thermal, and mechanical stability compared to organic polymers. This transfer method allows to access the fabricating for highly uniform van der Waals heterostructure devices, with performance limitations determined solely by the intrinsic quality and dimensions of the 2D films.

### 2.3 Quasi-Dry Transfer

The quasi-dry transfer method has garnered considerable attention, particularly in transferring 2D films, especially in scenarios where layer-by-layer splitting and seamless assembly of materials are required to fashion intricate 2D heterostructure. Combining the merits of wet and dry transfer method, this approach amalgamates the essential elements for a transfer process that minimizes damage, mitigates cracks, and curbs contaminations. A brief table for the latest outcomes on the quasi-dry transfer strategies of 2D films and associated applications is addressed in Table 3.

**Table 3:** Quasi-dry Transfer Strategies on Representative 2D Films and Related Applications^a^

2D Films	Support layer	2D Films Size	Devices	ref
MoS2	PMMA, and PDMS	–	FETs	(155)
WS_2_	PDMS	–	Photodetector	(156)
BP	PDMS/PC stamp	–	Optical Application	(157)
MoS_2_	PDMS	–	Photodetector	(158)
MoS_2_	PDMS	1.3 cm^2^	Photodiode	(159)
Graphene	Bisbenzocyclobutene (BCB)	–	Field-effect device	(160)
WS_2_, WSe_2_, graphene, h-BN, MoSe_2_, MoS_2_,	Ni-assisted transfer	5 cm	FETs	(161)

a Here, “–” means “not applicable”.

**Figure 17:**
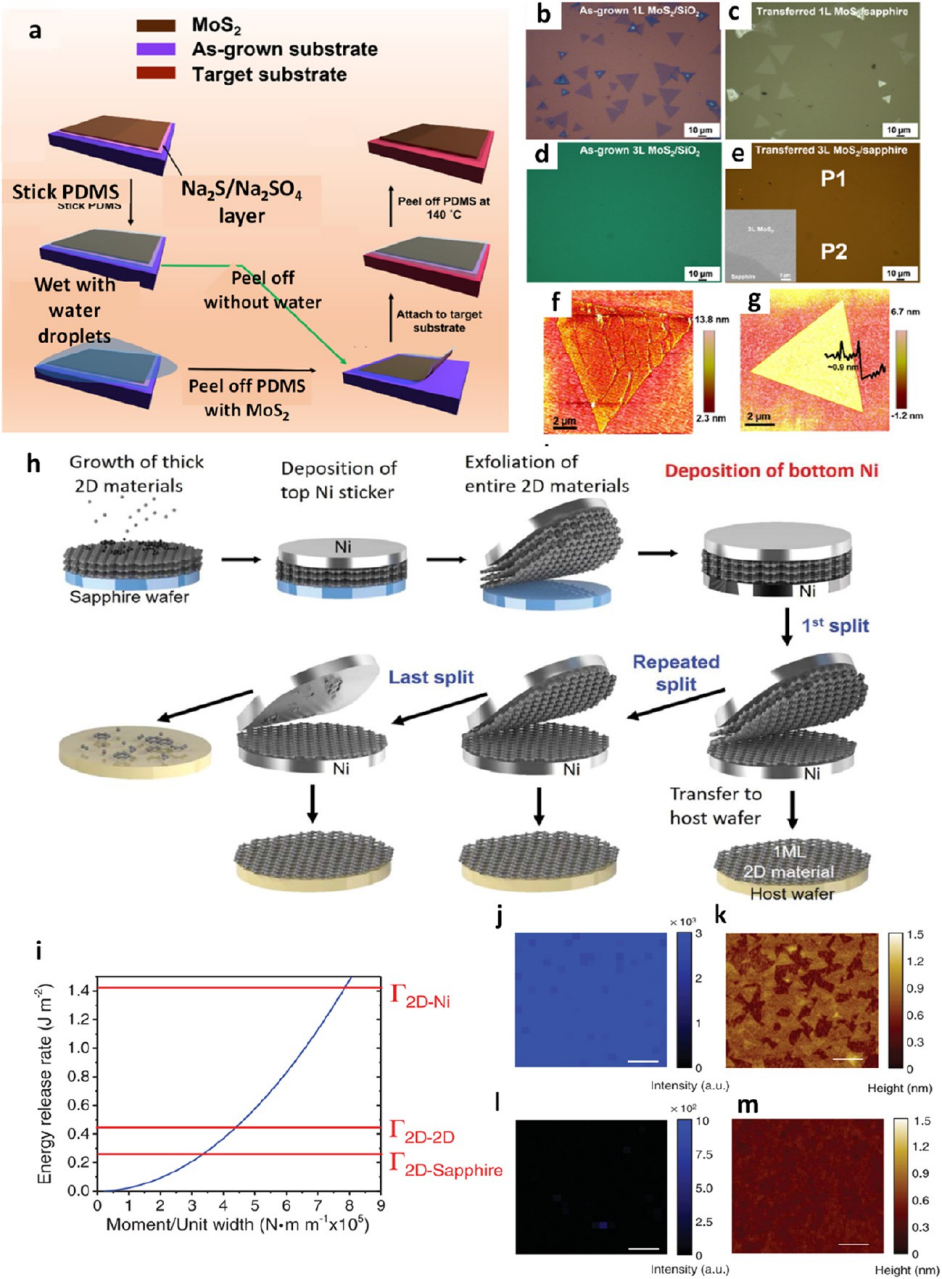
Quasi-dry transfer of MoS_2_ and WS_2_. a) Step-by-step schematic of the quasi-dry transfer process. b, c) monolayer MoS_2_ and d, e) trilayer MoS_2_ from SiO_2_/Si to the sapphire substrate. f, g) AFM topographical images of transferred monolayer MoS_2_ on the sapphire substrate without and with water, respectively. a-g) Reproduced with permission from ref (158). Copyright 2022 American Chemical Society. h) and (i) A representative method for transferring 2D heterostructures on a large scale, utilizing wafer-sized dimensions. Reprinted with permission under a Creative Commons (CC 4.0) License from ref (160). Copyright 2021 Nature Publishing Group. j) Schematic illustration explaining the transfer process for 2D films. k) Modeling of energy release rate according to applied moment. l) Mapping the intensity of Raman signals from the WS_2_ layer. m) AFM topography captured from the surface of as-grown WS_2_, which is 4 nm thick on the sapphire wafer. n) Raman mapping image illustrating the WS_2_ on sapphire substrate after postexfoliation of the WS_2_ layer. o) AFM topology obtained from the underside of WS_2_ layer after exfoliation h-n) Reproduced with permission from ref (161). Copyright 2019 AAAS.

For instance, in a study by Sharma et al., the quasi-dry transfer method was employed to transfer MoS_2_ onto the target substrate after its growth.^158^ The process was meticulous: a PDMS film was initially affixed to the freshly grown MoS_2_ flakes/film. Subsequently, the PDMS/MoS_2_/growth substrate assembly was delicately exposed to deionized (DI) water droplets. With a gentle peeling motion, the PDMS/MoS_2_ stack was methodically separated from the growth substrate. Underneath the MoS_2_ layer, the Na_2_S/Na_2_SO_4_ stratum dissolved in the water, extending mechanical support through buoyancy and shielding the MoS_2_ film from damage during peeling. To avert water entrapment, the PDMS/MoS_2_ stack was carefully dried using nitrogen (N_2_) gas in preparation for the subsequent dry transfer step, culminating in the assembly’s placement onto the target substrate. Finally, the PDMS film relinquished its bond to the surface of the MoS_2_ flakes/film, facilitated by applying heat to the PDMS/MoS_2_/target substrate assembly on a hot plate. The PDMS, with its adhesive properties altered by the heat, readily separated from the target substrate (Figure 17a-g). In another innovative approach by Quellmalz et al., graphene has been transferred using an adhesive layer of Bisbenzocyclobutene (BCB), a thermosetting polymer, which is spin-coated onto the target wafer.^160^ Subsequently, a softbake is applied to remove solvent and solidify the adhesive layer. Following this, the 2D materials grown on the initial substrate are placed onto the target wafer such that they face the adhesive layer and are loaded into a commercial wafer bonder. Heating temporarily reduces the viscosity of the adhesive layer, while the bond chuck applies uniform force to the wafer stack. As a result, the adhesive layer conforms to the 2D material, forming a stable bond with the target wafer and replicating the surface topography of the growth substrate without exerting excessive pressure on the 2D material. This characteristic is advantageous as it minimizes the risk of damage, wrinkles, or strain in the transferred 2D materials (see Figure 17h, (i).

While Shim et al. demonstrated an exceptionally versatile approach. They facilitated the transfer of WS_2_ and permitted the sequential detachment of individual WS_2_ layers. This development holds tremendous significance, especially in precisely controlling WS_2_ thickness for specific applications. After removing the top Ni/WS_2_ stack from the sapphire substrate, a bottom deposition layer of Ni was introduced through thermal evaporation and subsequently transferred to the target substrate (Figure 17j). The interfacial toughness represented as Γ_2D-Sapphire_ was lower than Γ_2D-Ni_ (Figure 17k). Consequently, the exfoliation of Ni/2D film stacks allowed for separating the weakest 2D-sapphire interface, thereby ensuring the clean detachment of 2D films from the wafer. In other words, it becomes entirely feasible to isolate and control each layer of 2D films by depositing top and bottom Ni layers onto the 2D films. In the initial detachment phase to separate the Ni/WS_2_ stack from the sapphire, the WS_2_-sapphire interface, being the most vulnerable, led to the full release of the WS_2_ layer from the substrate. Following exfoliation, no traces of WS_2_ were detected through Raman mapping on the sapphire wafer, indicating the flawless release (Figure 17-o). By strategically applying a pivotal force during the liftoff procedure, sufficient strain energy was imparted to the Ni/WS_2_ stacks, causing the delamination of the most vulnerable 2D-sapphire interface. This successful delamination led to the pristine release of the entire WS_2_ film, preserving the integrity of the underlying WS_2_ layers. The bottom WS_2_ layer displayed a continuous and smooth profile, characterized by a root-mean-square roughness of 0.5 nm, resulting from the seamless nuclei merging from the initial layers. Furthermore, to acquire a monolayer of WS_2_, a secondary detachment procedure was implemented by depositing a Ni layer onto the underside of the WS_2_ film while preserving the upper tape/Ni/WS_2_ stack postexfoliation. Given the considerably elevated interfacial energy (Γ_2D-Ni_ ∼ 1.4 Jm^2–^) reported between the Ni film and 2D films, surpassing 3-fold the van der Waals interaction within 2D films (Γ_2D-2D_ = 0.45 Jm^2–^), cracks emerged in proximity to the lower Ni layer and propagated through the comparatively weaker WS_2_–WS_2_ interface immediately above it. Consequently, the Ni/WS_2_ stack underwent separation during the peeling process, with the lower Ni layer strongly adhering to the WS_2_ monolayer, leaving behind an unblemished monolayer of WS_2_ atop the lower Ni layer.

This method proves to be a highly effective approach for transferring 2D films and for precise control of the desired thickness of these materials on the target substrate. It can seamlessly transfer and integrate various 2D films, resulting in 2D heterostructures with minimal contamination, thus ensuring a high purity level. This high-throughput manufacturing of 2D films is poised to serve as the cornerstone for commercializing devices based on 2D films.

## 3 The Mechanism Affecting the Quality of 2D Film Transfers

**Figure 18:**
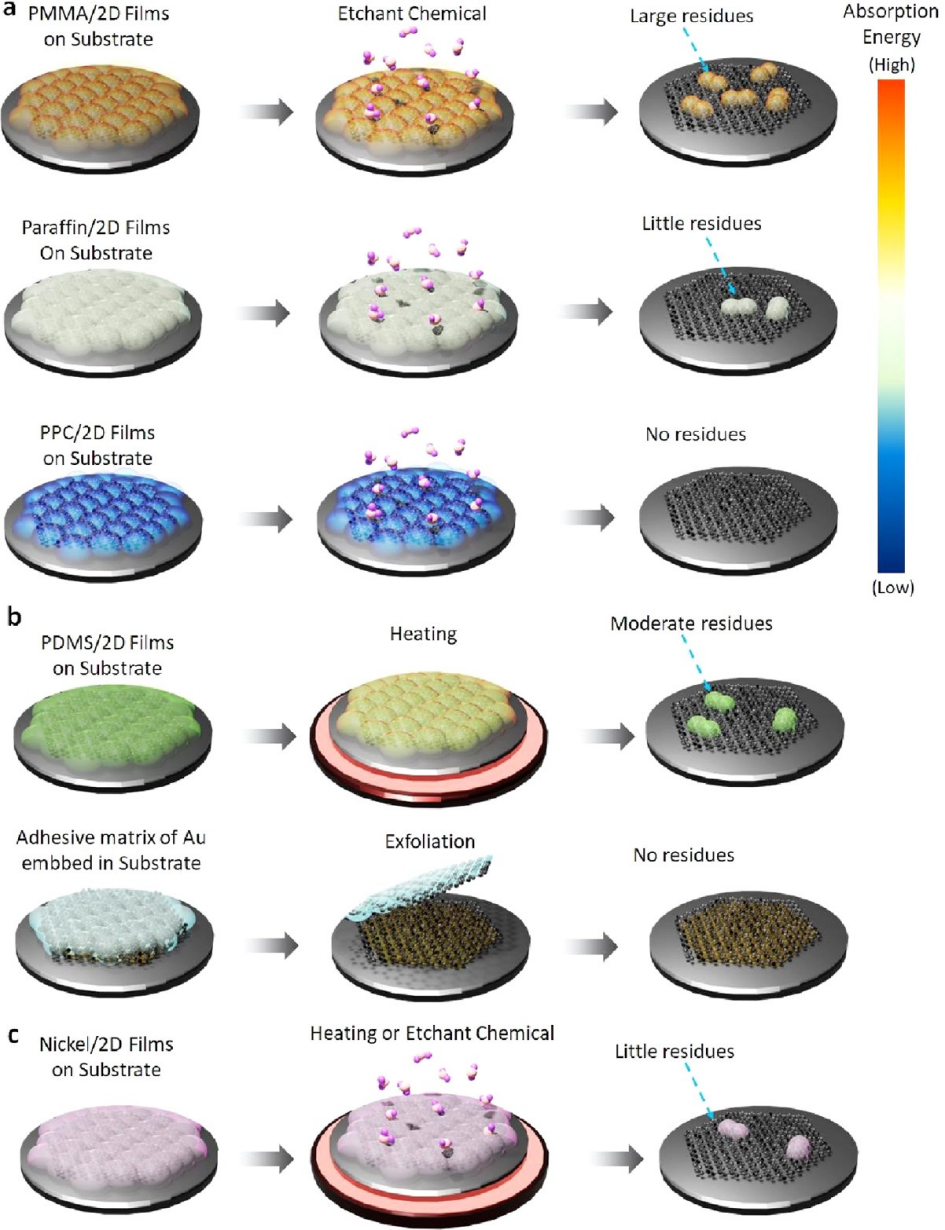
Models depict the possible mechanisms arising from removing the transfer support layer via different transfer strategies for 2D films composed of a) wet transfer method employing PMMA, paraffin, and PPC, highlighting the variations in absorption energy between the support transfer layer and 2D films; b) dry transfer method utilizing PDMS, and adhesive matrix of Au embed in substrate-assisted transfer, and c) quasi-dry transfer employing a nickel layer.

To assess the efficacy of a transfer method, the ultimate quality of 2D material remains a critical factor in determining the method’s capabilities. Since each transfer mechanism operates differently, the impact on 2D films varies across methods. This underscores the necessity of comprehending the transfer process’s working mechanisms and stepwise progression. Additionally, anticipating potential contamination throughout the transfer process is crucial for preventing, optimizing, and ensuring that the quality of 2D films is maintained before and after transfer, preserving their original properties and morphology. Building on the reviewed reported in the preceding section, this segment aims to identify and discuss the primary factors and influences that may lead to discrepancies on the surface of 2D films for each transfer method. Figure 18 illustrates the transfer support layer and chemical and temperature factors in the transfer mechanisms affecting 2D films.

### 3.1 Influence of the Transfer Support Layer

As discussed in previous sections of typical transfer methods, a layer of material, which can be either a polymer or metal, is used to cover and facilitate the transfer process of 2D films. However, suppose external factors such as temperature, pressure, chemicals, etc., are overlooked; another factor that can significantly impact material quality stems from the intrinsic surface energies at the interface of 2D films and the support layer. For instance, cellulose acetate (CA) has recently been integrated and utilized as an alternative supporting layer for the transfer processes of 2D films.^162^ While CA films can be dissolved by acetone, leaving considerably less residue than PMMA, direct contact between CA and 2D films has shown potential damage to the material surface, such as cracks and holes in the transferred film. As reported by Shim et al., layer-by-layer separation of 2D films can occur based on the difference in energy release rates between the support layer of Ni and 2D films.^161^ The authors successfully separated monolayers of 2D films at the wafer scale from multilayers, facilitating the integration of 2D films into semiconductor devices on a wafer-scale. In addition, the special properties of materials used for the support layer, such as paraffin and PPC, which possess a high thermal expansion coefficient and high Young’s modulus, respectively, are crucial considerations.^163,164^ Consequently, the material surface’s wrinkles can be significantly reduced throughout the transfer process. Moreover, to comprehend why residues of paraffin and PPC on the surface of 2D films are minimal, calculations based on the density functional theory (DFT) have been undertaken. Specifically, for paraffin, Leong et al. noted that the carbonyl groups of PMMA dimer are highly reactive compared to the rest of the molecule. In contrast, the reactivity of paraffin is generally uniform throughout the molecule.^56^ Additionally, adsorption energies indicate that PMMA adheres more strongly to the surface of graphene than paraffin. Thus, the formation of the paraffin-graphene interface is primarily driven by noncovalent interactions, and no covalent bonds are formed between paraffin and graphene. Even for PPC, Mondal et al. observed that the carbonyl groups exhibit even lower absorption, reaching approximately 91 meV per cell.^69^ These findings demonstrate that paraffin and PPC may serve as superior transfer support layers, resulting in fewer residues and ensuring the integrity of 2D films after the transfer process. From that, examining and determining the materials that support the transfer process of 2D films is a critical task in comprehending and understanding the enhanced mechanisms of 2D films in semiconductor devices.

### 3.2 Influence of Chemical

The chemical impact is most evident in the wet and quasi-dry transfer methods, where the substrate or support layer removal step often involves aqueous solutions for etching. In a 2014 report, Seifert et al. observed that electrochemical-assisted graphene transfer, with increased NaOH concentration, resulted in a smoother detachment of PMMA/graphene from the substrate. However, excessively high concentrations could also chemically affect the film quality, potentially causing cracks on the material surface.^165^ To elucidate the effects of etchants on the transfer process further, Wang et al. transferred graphene to SiO_2_/Si substrates using various solutions to remove, noting that FeCl_3_ and Cu etchants, both containing Fe^3+^ as the active ingredient, produced more continuous graphene morphologies with fewer cracks and holes.^166^ On the other hand, the graphene transfer process with nitric acid (HNO_3_) resulted in many holes due to nitrogen dioxide (NO_2_) bubbles generated during etching.

Similar results were observed with ammonium persulfate ((NH_4_)S_2_O_8_), leaving residue spots on the graphene surface under optical microscopy due to its strong oxidizing capacity. This observation stems from the notable oxidizing potential of (NH_4_)S_2_O_8_ with a standard electron potential nearly three times higher than Fe^3+^. Consequently, (NH_4_)S_2_O_8_ can oxidize and damage the protective PMMA layer, forming cracks and holes. In contrast, Fe^3+^-based solutions and commercial Cu etchant, which include a wetting antifoam agent, provide a milder and safer etch rate, enhancing integrity and reducing polymer residues. The impact of chemicals on the quality of 2D films is significant, potentially influencing the structure and morphology of 2D films, leading to deviations and hindered performance in integrating 2D films into devices. Therefore, carefully selecting and evaluating the quality of the chemicals involved in transfer processes is crucial. Achieving 2D films with minimal damage, cracks, and wrinkles post-transfer remains a top priority for any application.

## 4 The Best Cleanliness Transfers-Assisted Applications

Based on each transfer method’s distinctive features and advantages, numerous 2D films have successfully been integrated into electronic and optoelectronic devices in various ways.^86,167,168^ Several reports have demonstrated that transfer-assisted methods, such as wet-transfer, dry-transfer, or quasi-dry transfer, have yielded promising results and maintained high-quality 2D films post-transfer.^169−171^ For instance, in the case of wet-transfer, Kim et al. successfully transferred and integrated graphene into FETs without affecting the intrinsic characteristics of graphene.^172^ The resulting flexible graphene FETs operated at a low supply voltage of 4 V and exhibited almost negligible changes in I–V characteristics after 5000 bending and releasing repetitions with a 0.5 cm bending radius. In another application involving dye-sensitized solar cells (DSSCs), Arhin et al. employed an electrochemical method to transfer graphene in 1 M NaOH solution.^173^ High crystallinity was observed and confirmed through Raman spectroscopy and SEM images. Moreover, the measurement of short-circuit current density (*J*_sc_), open-circuit voltage (*V*_oc_), fill factor (FF), and overall conversion efficiency under AM 1.5, 100 mW cm^–2^ illumination yielded a value of 12.7 mA/cm^2^, 544.8 mV, 57.5%, and 3.8%, respectively. Many other reports have showcased a diverse range of applications for transferring and integrating 2D films. However, this article will focus on and discuss applications related to three transfer methods that we consider among the best with minimal impact on device performance and quality. These methods include Paraffin-assisted transfer, PPC-assisted transfer, and Quasi-dry transfer.

### 4.1 Paraffin-Assisted Transfer for FETs and Photodetectors

**Figure 19:**
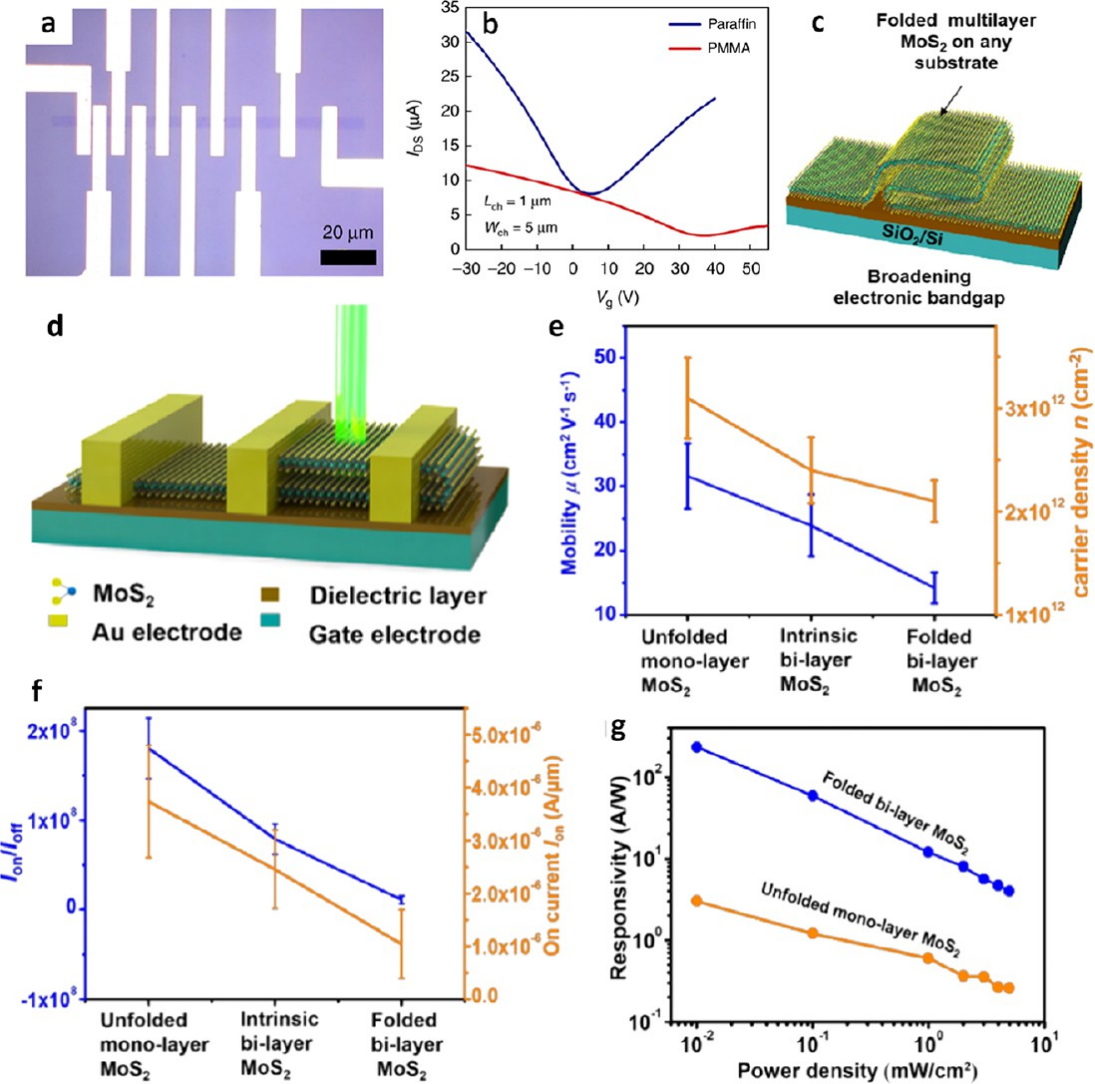
Paraffin-assisted transfer graphene and MoS_2_ for FETs and photodetectors. a) Visual representation of a standard array of graphene FETs with progressively lengthening channel lengths. b) A comparison of the transfer characteristics of two FETs manufactured on paraffin-transferred graphene reveals a significantly reduced and closer-to-zero magnitude. a-b) Reprinted with permission under a Creative Commons (CC BY 4.0) License from ref (56). Copyright 2019 Nature Publishing Group. c) Schematic illustration of folded multilayer MoS_2_ by PCF. d) Schematic diagram of the FETs and photodetectors of folded MoS_2_ using PCF strategy. e) Mobility μ, carrier density n, and f) I_on_/I_off_ on current I_on_ of unfolded monolayer, intrinsic bilayer, and folded bilayer MoS_2_. g) Responsivity and power density of unfolded and folded MoS_2_ at V_ds_ = 1 V and V_gs_ = 0 V. c-g) Reproduced with permission from ref (174). Copyright 2021 American Chemical Society.

As mentioned in section 2.1.1., paraffin serves as a polymer support layer for material transfer. It mitigates wrinkles in graphene arising from the thermal expansion of paraffin, which translates into tensile strain on the underlying graphene film. To assess the electrical performance of devices created with paraffin-transferred graphene, more than 100 graphene back-gate FETs were produced on Si/SiO_2_ substrates and subsequently underwent testing (Figure 19a).^56^ The electrical measurements were carried out at room temperature in ambient conditions, comparing the characteristics of FETs constructed with PMMA-transferred and paraffin-transferred graphene (Figure 19b). Following these examinations, the determined hole and electron mobilities for paraffin-transferred graphene were 14.215 and 7.438 cm^2^ V^1^s^–1^, respectively. In contrast, PMMA-transferred graphene exhibited notably lower hole and electron mobilities at 3.719 and 1.653 cm^2^ V^–1^s^–1^. The data clearly shows that the transfer of graphene using paraffin substantially boosts the effective hole and electron mobility, increasing them by a factor of 3.8 and 4.5, respectively. The improvement can be ascribed to decreased charge carrier scattering centers in the graphene transferred with paraffin. Similarly, Zeng et al. demonstrated that paraffin could influence the band gap of MoS_2_.^174^ By precisely controlling the temperature variations during paraffin-enabled compressive folding (PCF), they introduced controlled compressive strain ranging from 0.2 to 1.3%. This resulted in folded structures with an adjustable increase in band gap on various substrates (Figure 19c). As a result, this method allowed the creation of FETs and photodetectors with enhanced performance, featuring increased mobility and photoresponsivity. Through a statistical analysis of 30 FETs, the mobility values for folded bilayer, intrinsic bilayer, and unfolded monolayer MoS_2_ were determined to be 32.4 ± 4.7 cm^2^ V^–1^s^–1^, 24.3 ± 4.1 cm^2^ V^–1^s^–1^, and 32.4 ± 4.7 cm^2^ V^–1^s^–1^, respectively, while the carrier density was (3.2 ± 0.4) × 10^12^ cm^–2^_,_ (2.7 ± 0.4) × 10^12^ cm^–2^, and (2.4 ± 0.2) × 10^12^ cm^–2^ (Figure 19d-f). Additionally, the photoresponsivity versus power density of folded and unfolded MoS_2_ were compared (Figure 19g). In this context, it was demonstrated that the photoresponsivity of folded MoS_2_ was nearly 20 times higher than that of unfolded MoS_2_. The increased photoresponsivity observed in folded MoS_2_ primarily stems from amplified light absorption, as evidenced by the intensified photoluminescence (PL) spectra. It also indicates that employing the PCF approach to create folded multilayers improves optoelectronic performance.

The promise and potential of this technique could be further extended into various applications, where paraffin facilitates the transfer of large-area 2D films while preserving their intrinsic properties. Moreover, leveraging paraffin’s thermal properties, this approach minimizes surface wrinkles in 2D films and reduces the levels of polymer contamination. Paraffin’s low chemical reactivity and limited noncovalent affinity to 2D films are key to achieving these benefits. All these factors indicate that this is a versatile method that avoids unwanted contamination and enables easier integration into high-precision electronic and optoelectronic devices.

### 4.2 PPC-Assisted Transfer for Solar Cells and Light-Emitting Diodes (LED)

**Figure 20:**
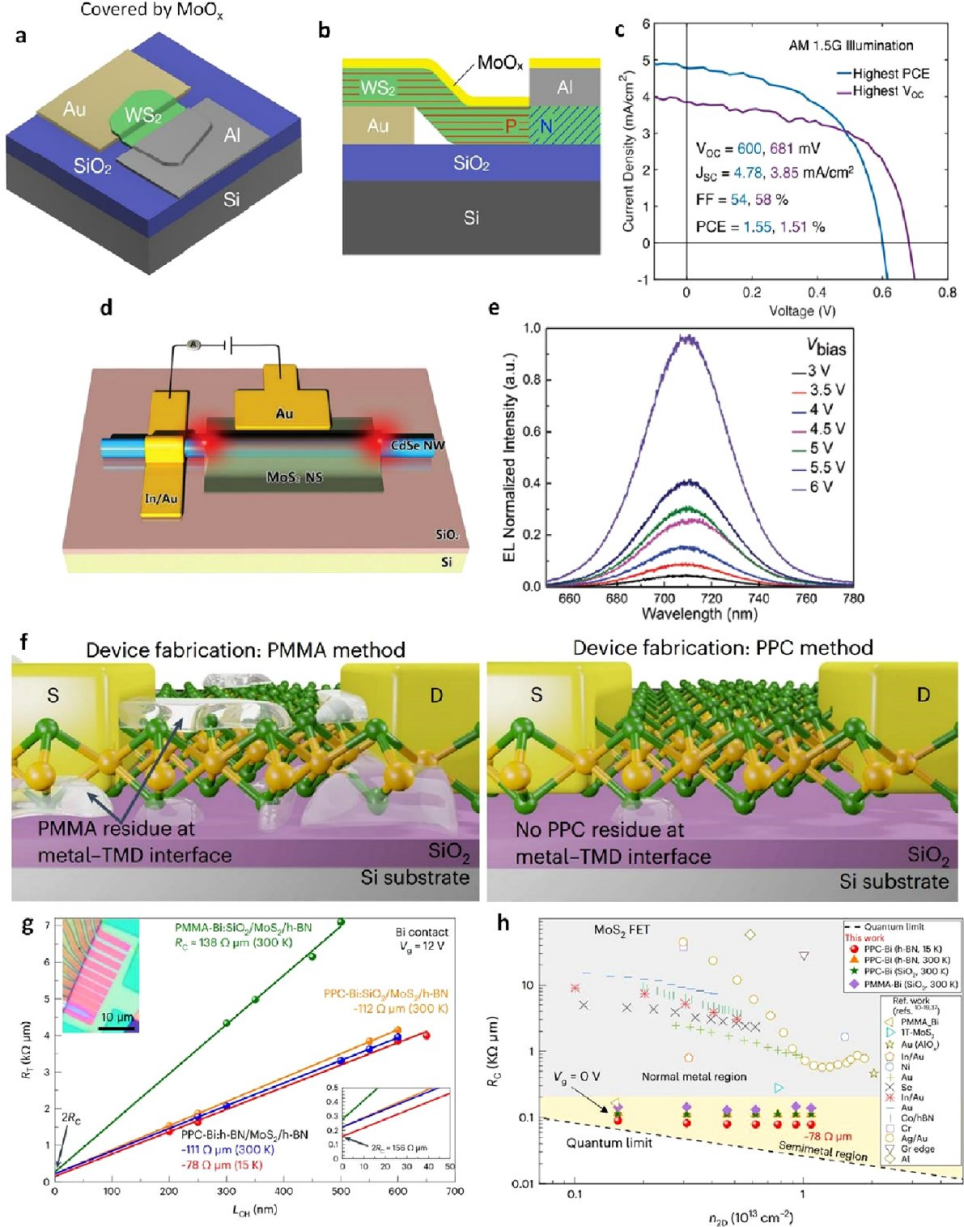
PPC-assisted transfer WS_2_ and MoS_2_ for Solar Cells, LED, and FETs. a) Schematic illustrates the lateral p–n junction multilayer WS_2_ solar cells; the device is covered by MoO_*x*_ (not shown). b) Cross-sectional view of the device. Reproduced with permission from ref (177). Copyright 2021 American Chemical Society. d) Schematic illustration of a mixed-dimensional LED based on the p-type MoS_2_ and n-type CdSe NW. e) The EL spectra of the heterojunction LED at various forward biases. Reproduced with permission from ref (178). Copyright 2017 Royal Society of Chemistry. f) The top panel illustrates the schematic model of a MoS_2_ FET device fabricated using PMMA, showcasing residues at the metal-MoS_2_ interface (indicated by arrows). This PMMA method introduces residues that negatively impact device performance. In contrast, the bottom panel depicts the PPC method, which exhibits minimal residues, leading to improved device performance by eliminating transfer process-induced residue. The labels S and D represent the source and drain, respectively. g) The extracted R_c_ value (2R_c_ = y-intercept of R_T_, total resistance) for Bicontact devices are shown using the transfer-length method for device 1 (in red and blue), device 2 (in orange), and device 4 (in green). The top inset displays the device image, while the magnified y-intercepts (2R_c_, in the bottom inset) highlight the lowest R_c_ value (∼78 Ωμm) observed device 1 at 15 K. L_CH_, channel length. h) A benchmark comparison of Rc versus n_2D_ in MoS_2_ FETs using different metal contacts for various semiconductor technologies. The 1T-MoS_2_ represents the 1T phase of MoS_2_, and Gr stands for graphene. f-h) Reproduced with permission from ref (69). Copyright 2023 Nature Publishing Group.

The PPC is highly regarded as a safe and nontoxic material with strong adhesion properties and low chemical reactivity toward 2D films.^175,176^ These attributes are of paramount importance in applications within electronics and optoelectronics, where concerns over contamination and toxicity loom large. For instance, in the context of enhancing the performance of solar cells based on p–n junction of transition metal dichalcogenides (TMDs), enabled by MoO_*x*_ doping and passivation, meticulous transfer of WS_2_ flakes from development substrates to Si/SiO_2_ substrates using PPC as a transfer support layer in order to construct a WS_2_/MoO_*x*_ solar cells structure alongside Au and Al electrodes (Figure 20a, b).^177^ Notably, the J-V measurements groundbreaking open-circuit voltage (*V*_oc_) of 681 mV, leading to a record-high power conversion efficiency (PCE) of 1.55% in ultrathin WS_2_ photovoltaic cells. The highest device exhibits a short-circuit current density (*J*_sc_) of 4.78 mA/cm^2^, along with a reasonable fill factor (FF) of 54% (Figure 20c).

Similarly, another study involves a 2*D*/1D van der Waals LED based on p-type MoS_2_ and n-type cadmium selenide (CdSe) nanowire (NW) junction (Figure 20d).^178^ Here, PPC plays a pivotal role. After synthesizing CdSe NW on Si/SiO_2_ substrate, MoS_2_ is transferred onto the surface of CdSe NW with PPC acting as a supporting layer for the transfer process. Subsequently, the PPC layer is removed through a brief heating process at 90 °C for 1 min. The performance of this LED is demonstrated in part through the measurement of electroluminescence (EL) spectra at various forward biases, and the relationship between EL intensities and input power is presented (Figure 20e). It is worth noting that the MoS_2_ transfer process using PPC has no adverse effects on the quality or morphology of MoS_2_, ensuring that the device structure is maintained and capable of achieving optimal performance. To elucidate the impact of PPC on the manufacturing process and performance measurement of devices, Mondal et al. ingeniously incorporated MoS_2_ into FET devices using PMMA and PPC (Figure 20f).

The authors fabricated five distinct types of devices for comparison: (i) PPC-transferred Weyl semimetal Bicontact FET on h-BN substrate (device 1, PPC-Bi:h-BN/MoS_2_/hBN), (ii) the same configuration on SiO_2_ substrate (device 2, PPC-Bi:SiO_2_/MoS_2_/h-BN), (iii) PPC-transferred Ti-contact FET on SiO_2_ substrate (device 3, PPC/TiO_2_:SiO_2_/MoS_2_/h-BN), (iv) PMMA-transferred Bi contact FET on SiO_2_ substrate (device 4, PMMA-Bi:SiO_2_/MoS_2_/h-BN), and (v) PMMA-transferred Ti-contact FET on SiO2 substrate (device 5, PMMA-Ti:SiO_2_/MoS_2_/h-BN). In device 1, the contact resistance is approximately 78 Ωμm at 15 K, lower than the ∼92 Ωμm in device 2 with SiO2. At room temperature, the contact resistance in device 1 slightly increases to ∼111 Ωμm, which is the lowest among all devices. A similar trend is observed with Vg = 0 V, although the contact resistance of all devices is slightly elevated due to the low carrier density at Vg = 0 V. This unusually low contact resistance is attributed to a residue-free interfacial contact between the Weyl semimetal Bi and MoS_2_, effectively overcoming Fermi-level pinning. Additionally, the use of an h-BN substrate minimizes substrate scattering. Referring to Figure 20h, it is evident that the benchmark with state-of-art R_c_-n_2D_ values (n_2D_, 2D carrier density) for various metal contacts used in semiconductor technologies showcases the superiority of the Bi/MoS_2_ FET (device 1). This device exhibits the lowest R_c_ (∼78 Ωμm) at a 2D carrier density, n_2D_ = 1.1 × 10^13^ cm^–2^ at 15 K. The contact resistance slightly decreases at higher n_2D_ values. Notably, all Bicontact devices show significantly lower R_c_ than conventional metal contacts used in this study (Ti) and the literature. At zero Vg (n_2D_ = 1.5 × 10^12^ cm^–2^), R_c_ approaches the theoretical quantum limit of 66 Ω μm (Figure 19h)

PPC stands out as an exceptional material for transfers because of its inherent sensitivity to temperature. At low temperatures, it exhibits strong adhesion to 2D films. However, at higher temperatures, the adhesion between 2D films and PPC significantly weakens, enabling 2D films to be easily released from the PPC film and transferred with minimal residue. This method is also highly controllable and contributes well to integrating 2D films into semiconductor devices.

### 4.3 Adhesive Matrix-Assisted for Transistors

**Figure 21:**
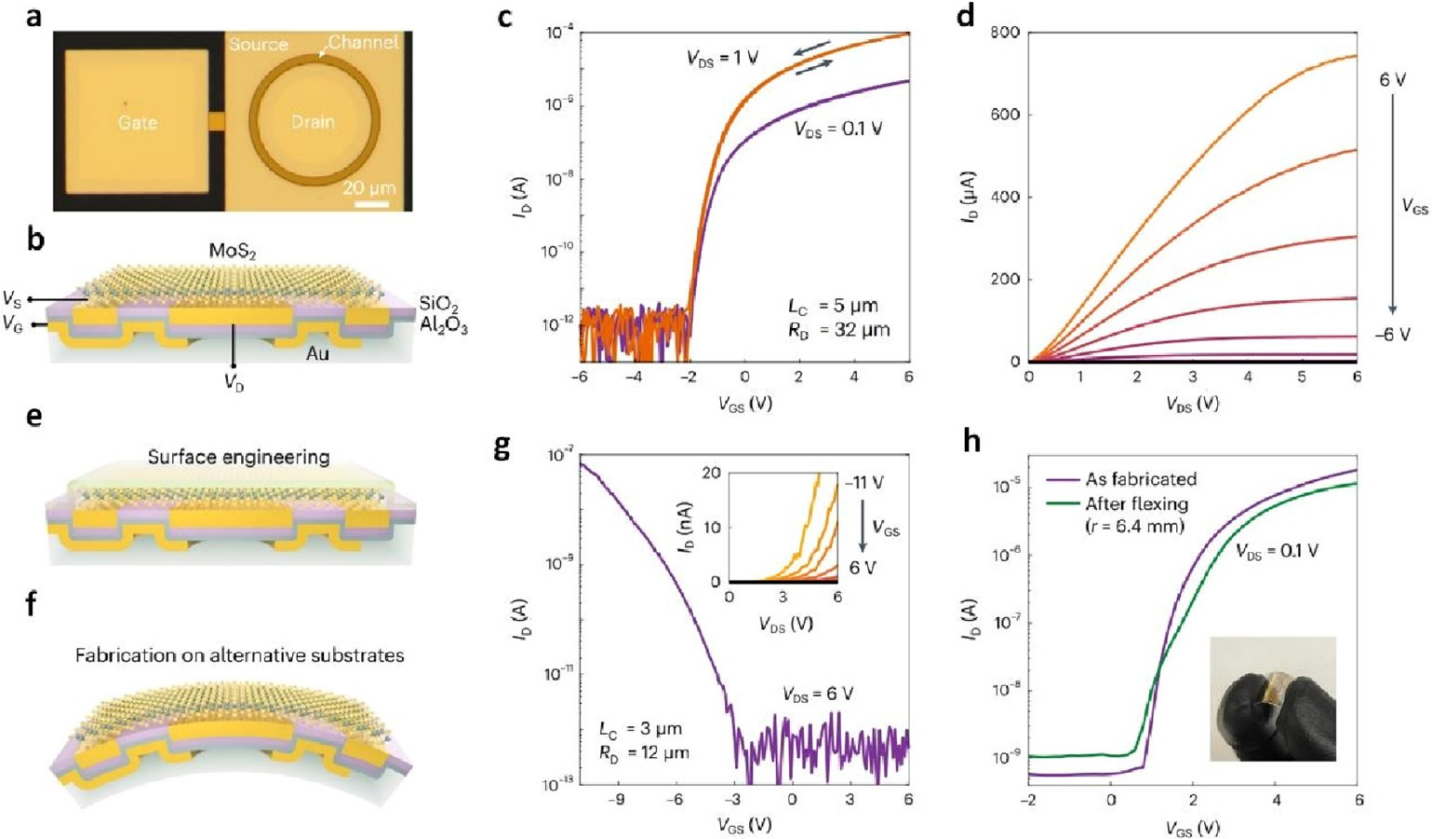
Adhesive matrix–assisted transfer MoS_2_ for transistors. Optical micrograph a) and schematic cross-section b) depict a transistor that has been successfully fabricated. In this configuration, the channel length (L_C_) measures 5 μm, and the drain radius is 32 μm. c) transfer characteristic (drain current I_D_ versus gate-source voltage) of the aforementioned transistor (a) reveals a significant on–off ratio (∼108), minimal hysteresis (<300 mV), and favorable subthreshold swing (202 mV dec^–1^). d) output characteristic (I_D_ versus drain-source voltage V_DS_) is presented for V_GS_ ranging from 6 to −6 V. e) Schematic illustration underscores the potential for surface engineering facilitated by creating a pristine device with a clean exposed surface. f) Another schematic depicts a device crafted on a flexible substrate, made possible by our innovative electrode fabrication approach. g) Electrical characterization of a p-type monolayer-MoS_2_ device, achieved through AuCl_3_ charge-transfer doping, is displayed. The main plot exhibits the transfer characteristic, demonstrating a high on–off ratio (>104) and unipolar p-type behavior. The inset illustrates a nonlinear output characteristic attributed to the Schottky barrier for holes. h) transfer characteristic of a device on a PET substrate is shown before and after flexing to a radius of 6.4 mm. The inset includes a photograph of the fabricated flexible devices, with the substrate measuring approximately ∼9 mm in size. Reproduced with permission from ref (119). Copyright 2023 Nature Publishing Group.

By surpassing the constraints imposed by van der Waals interactions, adhesive matrix transfer emerges as a groundbreaking method for seamlessly integrating 2D films into devices in a single step. This innovative approach eliminates the need for post-transfer fabrication, solvent exposure, sacrificial layers, and high temperatures. Even after integration into electronic and optoelectronic devices, the surfaces of the 2D films maintain their pristine properties. In a study by Satterthwaite et al., the transfer process was demonstrated by fabricating a series of MoS_2_ monolayers applied in transistors (Figure 21a, b).^119^ Here, the adhesive matrix of Au in the substrate not only serves as a supportive layer for the detachment of MoS_2_ but also functions as the device’s source and drain contacts. Electrical characterization of the device revealed an on–off ratio of ∼10^8^ and an on-state current of ∼3.7 μA μm^–1^ (Figure 21c, d), showcasing n-type behavior with threshold voltages for the measured devices. Beyond ensuring a clean interface, this platform keeps the top surface of the 2D film pristine, enabling efficient customization of device performance (Figure 21e, f). Specifically, the surface above the 2D/metal contacts is available for further engineering. The author demonstrates that, through charge-transfer doping with gold chloride (AuCl_3_), the engineered n-type transistors can be manipulated to exhibit p-type behavior. Devices fabricated through this method display pure p-type behavior with no n-type branch and an off ratio exceeding 10^4^ (Figure 21g). Furthermore, the performance of these p-type devices can be fine-tuned by combining this doping approach with high-work-function metal adhesive matrices.

In contrast to existing approaches that require the direct fabrication of devices on the final flexible substrate, this strategy involves forming device features on a rigid carrier through conventional processing steps. The devices are then bonded and transferred to the final substrate, eliminating the need for process compatibility between the substrate and electrode fabrication procedure. This allows diverse substrate materials beyond commonly used polyimide films, enabling more efficient device integration. Figure 21h illustrates the electrical characterization of flexible transistors using this method.

This technique not only allows researchers to probe the intrinsic properties of the devices but also offers the flexibility to engineer devices with both n- and p-type behaviors. Additionally, adhesive matrix transfer is compatible with a range of substrates for flexible devices, expanding avenues to tackle challenges and optimize the potential performance achievable within constraints of 2D films.

4.4. Quasi-Dry Transfer-Assisted for Flexible Photodiode and Vertical Transistor This is a method that combines both wet and dry transfer techniques in the process of transferring 2D films. As a result, the advantages of each approach are harnessed effectively in this technique. To illustrate, consider employing PDMS as a supportive layer during the transfer procedure. PDMS and 2D films stacks can be effortlessly detached from the growth substrate in DI water due to the interaction between the hydrophobic 2D films/PDMS and the hydrophilic growth substrates.^179,180^ As water preferentially infiltrates between the 2D films and the growth substrate, it facilitates the surface-energy-driven removal of the 2D films. The disparity in surface energies encourages water molecules to permeate beneath the film, facilitating gentle detachment through water infiltration at room temperature, thereby eliminating mechanical forces from bubble formation and chemical etchant stacks. Conversely, when integrating 2D films onto target substrates, the PDMS layer can be completely eliminated via heating, ensuring minimal impact on the surface quality of the films. Therefore, this method offers a convenient solution for both transferring and integrating 2D films into devices, mitigating the potential for errors in the transfer process that could affect the quality and performance of the device.

**Figure 22:**
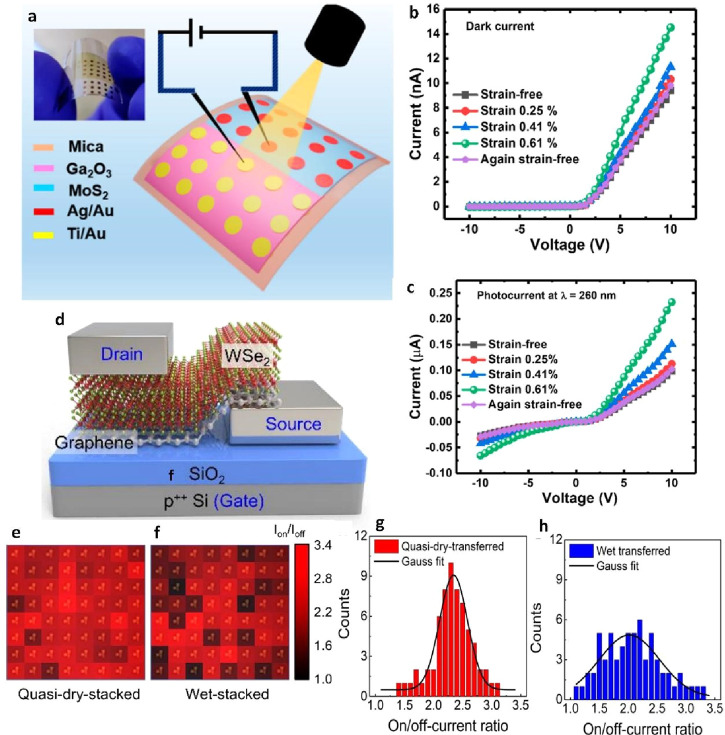
Quasi-dry-assisted transfer of MoS_2_, WSe_2_ and graphene for flexible photodiode and vertical transistor. a) Schematic illustration of the MoS_2_/Ga2O_3_ flexible photodiode. b) variation of dark current and c) photocurrent under different tensile strains. a-c) Reproduced with permission from ref (159). Copyright 2023 American Chemical Society. d) Schematic of graphene/WSe_2_ vertical transistor. e, f) 2D color maps of on/off-current ratio extracted from I_D_-V_G_ curves at V_DS_ = −0.5 V in vertical transistor arrays made by quasi-dry transfer (f) and wet-transfer (f) processes. g) Histograms of on/off-current ratios for 64 vertical transistors of quasi-dry-stacked and h) wet-stacked 2D heterostructures. d-h) Reproduced with permission from ref (161). Copyright 2018 AAAS.

In this manner, Sharma et al. achieved the transfer of MoS_2_ films onto the surface of Ga_2_O_3_, which had been synthesized on a flexible substrate, creating a heterojunction-based photodiode (Figure 22a).^159^ The authors obtained auspicious results, significantly enhancing the photoresponse through the piezophototronic effect. In this context, the electrical analysis of the heterojunction revealed exceptional photoresponse characteristics with an impressive phototo-dark current ratio of 10^3^. Furthermore, compared to a strain-free condition, the device exhibited a notable increase in photocurrent and responsivity by 155% and 136%, respectively (Figure 22b, c). In another report, the merits of the quasi-dry stacking process were further underscored by the fabrication of arrays of WSe_2_/graphene vertical transistors (Figure 22d).^156^ Through the deposition of Ni layers, these 2D films were extracted from sapphire wafers and subsequently delaminated layer-by-layer to reduce the thickness. This was accomplished by depositing a Ni layer beneath and eventually transferring them to the host substrate. The results revealed that in the realm of on–off ratios for these vertical transistors, the quasi-dry stacking process displayed outstanding device-to-device uniformity with only a 9.6% variation. The wet-stacking process exhibited a device-to-device variation of 26% (Figure 22e-h). These studies are a compelling testament to a highly promising avenue for transferring and integrating 2D films into electronic or flexible optoelectronic devices using quasi-dry transfer techniques.

## 5 Summary and Prospects

In summary all three major classes of transfer techniques, wet-transfer, dry-transfer, and quasi-dry transfer are valuable and widely used methods for transferring 2D films.^38,181−184^ An essential requirement for a successful transfer process is preserving the material’s structural integrity, minimize contamination, surface damage, and residue. These critical factors can be finely tuned through transfer conditions such as temperature, pressure, solution chemistry, environment, etc. Each transfer method has its unique advantages and disadvantages. For example, wet transfer is a mature technology with lower tensile stress but comes with drawbacks such as contamination, potential damage, time-consuming processes, and limitations on the transfer area. On the other hand, dry transfer offers high quality, larger transfer areas, and shorter processing times but is susceptible to tensile stress and residue. Recent advancements over the past decade have primarily revolved around improving the adhesion between the transfer support layers and 2D films using polymers with unique intrinsic characteristics like PPC and paraffin.^185−187^ The adhesion can be precisely controlled via temperature. Moreover, based on the thermal expansion coefficient, these polymers can help minimize wrinkles on the 2D film’s surface, significantly enhancing the quality and performance of 2D film when used in electronic and optoelectronic devices. Contrastingly, the adhesive matrix method refrains from utilizing any chemical etchants throughout the transfer process. Notably, the success of the transfer process relies heavily on the variance in adhesion between the 2D film and the adhesive matrix, as well as between different 2D layers. This approach strategically minimizes transfer steps, mitigating the risk of unwanted contaminants that may arise during the process. By directly peeling pristine 2D films from a substrate immersed in the adhesive matrix, the integration of 2D films into device substrates is achieved in a single step, preserving the intrinsic properties of the 2D films.

**Figure 23:**
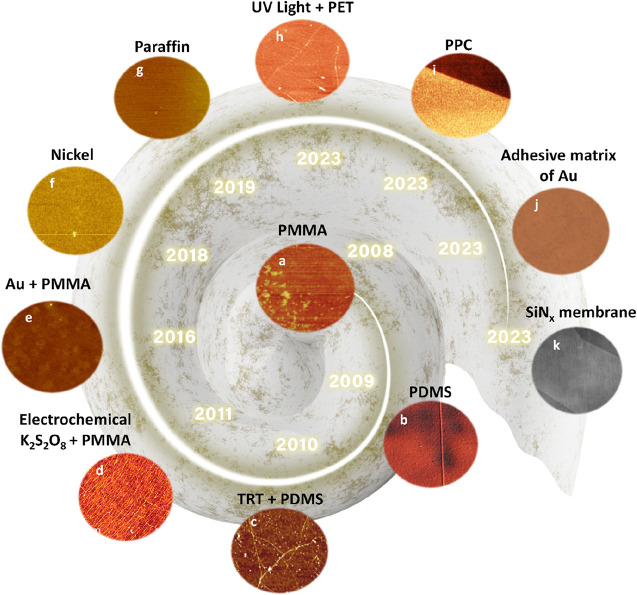
A model visually captures the evolution of transfer strategies from imperfection to perfection, spanning 2008 to 2023. AFM images vividly capture the surface quality of the 2D film after undergoing the transfer process via a) the use of PMMA. Reproduced with permission from ref (188). Copyright 2018 American Chemical Society. b) the use of PDMS. Reproduced with permission from ref (189). Copyright 2009 Willey-VCH. c) the use of TRT and PDMS. Reproduced with permission from ref (109). Copyright 2010 Nature Publishing Group. d) the use of electrochemical K_2_S_2_O_8_ solution and PMMA. Reproduced with permission from ref (190). Copyright 2011 American Chemical Society. e) the use of gold and PMMA. Reproduced with permission from ref (191). Copyright 2016 American Chemical Society. (f) The use a nickel layer. Reproduced with permission from ref (161). Copyright 2018 AAAS. (g) the use of paraffin. Reprinted with permission under a Creative Commons (CC BY 4.0) License from ref (56). Copyright 2019 Nature Publishing Group. h) the use of UV light and PET layer. Reproduced with permission from ref (142). Copyright 2023 American Chemical Society. (i) the use of PPC. Reproduced with permission from ref (69). Copyright 2023 Nature Publishing Group. j) the use of an adhesive matrix of Au. Reproduced with permission from ref (119). Copyright 2023 Nature Publishing Group. k) the use of SiNx membrane. Reprinted with permission under a Creative Commons (CC BY 4.0) License from ref (154). Copyright 2023 Nature Publishing Group.

Consequently, this represents a promising strategy for the future, offering a opportunity to harness the maximum performance of 2D films in electronic and optoelectronic devices. As another practical approach, leveraging the strengths of both wet-transfer and dry-transfer methods to develop a quasi-dry transfer technique has shown immense potential in transferring 2D film with minimal damage quality. Furthermore, it is relatively straightforward to delaminate 2D films layer-by-layer when using metal layers deposited above and below the 2D films. Variations in interfacial toughness between metal-2D, 2D-2D, and 2d-metal interfaces allow for precise layer-by-layer separation and subsequent transfer to the desired substrate. This is particularly significant in harnessing the unique properties of 2D films, such as their electrical and optical characteristics, whether in single-layer, bilayer, or trilayer configurations. Figure 23 shows the evolution of transfer methods, ranging from simple approaches to sophisticated and flawless techniques, minimally impacting the quality of 2D films. The initial transfer methods for 2D films, relying on PMMA support layers,^188^ often left significant residues that adversely affected the surface material quality. Subsequent improvements and enhanced transfer efficiency were achieved by utilizing polymers such as PDMS and TRT + PDMS.^109,189^ Ongoing innovation and diversification of various 2D film transfer approaches have continued through immersing PMMA/2D film/substrate into electrochemical systems with solutions like NaOH,^94^ CH_3_COOTBA,^96,97^ HNO_3_,^166^ (NH_4_)S_2_O_8_,^166^ and potassium persulfate (K_2_S_2_O_8_).^190^ Notably, the exploration of methods and techniques has persisted from 2016 until now, with the overarching goal of reaching a flawless method for defect-free and residue-free transfer of 2D films. For instance, using Au and Ni layers to enhance adhesion between them and 2D films facilitates easy detachment of 2D film layers from bare substrates.^161,191^ Another promising method involves employing UV light to remove support layers.^142^ Particularly in recent years, paraffin,^56^ PPC,^69^ adhesive matrix layers,^119^ and SiN_*x*_ membrane^154^ have emerged as outstanding strategies for 2D film transfer. These methods show minimal residues and negligible impact on the surface of 2D films post-transfer. As time progresses, transfer methods have evolved from imperfection to perfection. In the near future, the emphasis will likely shift toward simple, easy, and quality-assured transfer techniques that preserve the original intrinsic properties of 2D films. This ongoing investment and research aim to achieve perfection in transferring 2D films. In addition to transfer, equal extent of progress also needs to be made on another important and related subject which is synthesis of high quality 2D crystals on arbitrary substrates at low temperatures. This has been recently achieved either by selecting the 2D materials with lower growth temperatures like group III chalcogenides such as InSe or modifying growth systems. for TMDCs.

In addition, innovative transfer methods are being actively researched and developed, exemplified by an innovation by Liu et al.^192^ The authors introduced and demonstrated the efficacy of ice-aided transfer (IAT) and ice stamp transfer (IST) in successfully transferring various 2D films synthesized through CVD growth onto a wide array of substrates, including mica, sapphire, and SiO_2_. Furthermore, Zhang et al.’s diligent efforts have showcased a method known as water-transfer-printing (WTP) to transfer CVD-graphene onto arbitrary surfaces without relying on traditional polymer protections.^193^ Instead, the authors employed a liquid protection layer (LPL), modifications in the surface tension of the etchant, and the assistance of a low concentration of antiwrinkle agents (AWAs) that can be entirely removed. This method not only exhibits high feasibility and versatility in depositing onto a variety of surfaces but also results in a significant 100% enhancement in electrical performance compared to the conventional PMMA-assisted method, along with superior mechanical stability. These techniques hold great promise as potential solutions for bridging the gap between synthesizing 2D films and their practical applications. However, further research and validation are needed to ascertain the full spectrum of benefits and values these techniques can bring.

Beyond the realm of 2D films, the transfer, as mentioned earlier techniques, holds the potential for application across a wide spectrum of materials, such as perovskites,^194−196^ III–V semiconductor materials,^191−195,197−201^ and more. An example of transferring MAPbI_3_ and MAPbBrI_2_ for photovoltaic applications using PDMS layers was executed by Mohapatra et al.^202^ This approach demonstrates the versatility of the PDMS transfer method, facilitating the preparation of high-crystallinity perovskite materials with excellent surface coverage. This technique achieved notably PCEs of 14% and 7% for MAPbI_3_ and MAPbBrI_2_, respectively. In another instance, exploiting transfer techniques in photonic crystal-related applications, Liu et al. utilized PMMA layers to support the transfer of Au patterns.^203^ Notably, the Au connectors exhibited no signs of cracking, and the circuit’s performance remained unaffected after 1000 cycles of bending and releasing at nearly 180°. These methods can extend their application to various materials and objectives. Their flexibility and diversity make transfer techniques indispensable for seamlessly integrating materials into various electronic and optoelectronic devices.^204−211^

**Figure 24:**
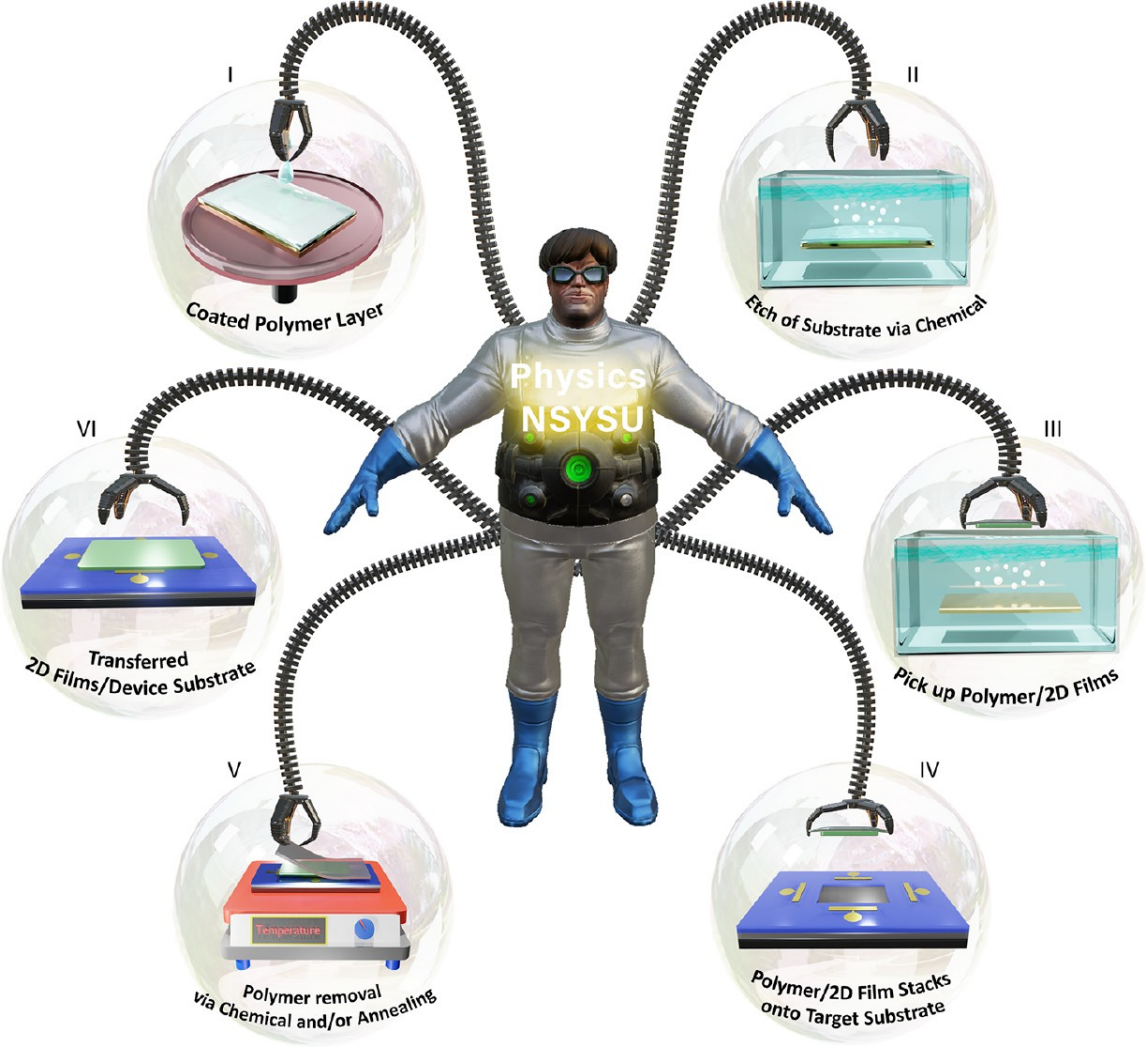
Visualization of an industrial mass-production integration of 2D film transfer via the lever-arm procedure imitated via Doctor Octopus in Spider-Man movie.

The ultimate objective in developing transfer methods of 2D layers is to minimize cracks and wrinkles in materials during the transfer process.^212,213^ This has been one of the most significant challenges to address thus far. Additionally, considering the interactions between the transfer support layers and the material is crucial, as each material exhibits unique interactions with these layers and with the development substrates and target substrates. Removing and cleaning residue are also distinct post-transfer challenges, as these residues must be eliminated to avoid undesired effects that could impact device performance. While current techniques have met the requirements to some extent, further research and development are needed to establish a comprehensive industrial process for mass production of 2D film transfer strategies poised to find widespread use in the industry. In particular, the challenge of maintaining the original properties, morphology, and quality of the materials pre and post transferring large-area 2D films remains a critical area of investigation in applications and the integration of 2D films into semiconducting devices. In Figure 24, a holistic overview of the promising applications of transfer methods is visualized as a pivotal step toward the future industrial-scale production of 2D films and their van der Waals heterostructure devices and circuits.

## Vocabulary

Thermal expansion coefficient: denotes how much a material expands as temperatures rise
Young’s modulus: is a mechanical property of solid materials that measures the tensile or compressive stiffness when the force is applied lengthwise
Binding Energy: amount of energy required to separate or to disperse a layer or material from a structure
Interfacial toughness: is the energy required to extend a pre-existing crack at an interface
Hydrophilic: are molecules or entities that are attracted to water molecules and tend to dissolve in water.
